# Mapping in-cell protein contact sites reveals hijacking of paraspeckles during influenza A virus infection

**DOI:** 10.1038/s41564-026-02416-1

**Published:** 2026-07-20

**Authors:** Iuliia Kotova, Lars Mühlberg, Konstantin Gilep, Mira Lea Burtscher, Isabelle Becher, Dingquan Yu, Daniel Ziemianowicz, Stephanie Stanelle-Bertram, Sebastian Beck, Kyungmin Baeg, Alexandra Grba, Olivier Duss, Gülşah Gabriel, Mikhail M. Savitski, Fan Liu, Boris Bogdanow, Jan Kosinski

**Affiliations:** 1 European Molecular Biology Laboratory Hamburg, Hamburg, Germany; 2 Centre for Structural Systems Biology (CSSB), Hamburg, Germany; 3 Department of Structural Biology, Leibniz-Forschungsinstitut für Molekulare Pharmakologie (FMP), Berlin, Germany; 4 Charité – Universitätsmedizin Berlin, Berlin, Germany; 5 Molecular Systems Biology Unit, European Molecular Biology Laboratory, Heidelberg, Germany; 6 Research Department Viral Zoonoses – One Health, Leibniz Institute of Virology (LIV), Hamburg, Germany; 7 Research Center for Emerging Infections and Zoonoses, University of Veterinary Medicine Hannover, Hannover, Germany; 8 Institute of Virology, Charité – Universitätsmedizin Berlin, corporate member of Freie Universität Berlin and Humboldt-Universität zu Berlin, Berlin, Germany

**Keywords:** Influenza virus, Long non-coding RNAs, Protein-protein interaction networks, Nuclear organization, Nucleus

## Abstract

Influenza A virus (IAV) hijacks host cellular machinery during infection but many host–virus protein interactions remain uncharacterized, particularly in their native context. Here, we applied in-cell cross-linking mass spectrometry, integrated with structural modelling and functional assays, to map protein–protein contact sites in IAV-infected human cells. This revealed previously unrecognized virus–host interactions linked to spatially organized processes. We identified host factors linked to the maturation of distinct glycoforms of the viral surface glycoprotein haemagglutinin through the membrane-bound endoplasmic reticulum–Golgi system. In the nucleus, we observed the progressive disassembly of paraspeckles (phase-separated membraneless compartments) across multiple cell lines. Mechanistically, viral nucleoprotein and non-structural protein 1 interact with host paraspeckle proteins, the viral endonuclease PA-X degrades long non-coding RNA housed within paraspeckles and viral RNA polymerase II is inhibited to drive paraspeckle disruption, which releases host factors that facilitate IAV replication. These findings uncover mechanisms by which IAV exploits and remodels host compartments during infection.

## Abstract (web summary)

In-cell cross-linking mass spectrometry maps protein contacts in influenza-infected human cells, revealing how the virus hijacks host membrane-trafficking factors and dismantles nuclear paraspeckles to enhance replication.

## Main

Influenza A virus (IAV) remains a global health threat^1^. Its replication relies on protein–protein interactions (PPIs) between up to 14 viral proteins^2^ and host factors, often confined to cellular compartments and organelles. Understanding these host–IAV PPIs in context is essential for elucidating viral strategies and therapeutic targets.

Viral RNA (vRNA) synthesis is confined to the nucleus and catalysed by the vRNA-dependent RNA polymerase complex (RdRp; PA, PB1 and PB2) within viral ribonucleoproteins (vRNPs), in which RdRp and vRNA wrap around nucleoprotein (NP) filaments^2^. Transcription and replication also depend on recruited host factors. Nuclear vRNA processing is modulated by context-specific PPIs between NS1 and NP and host RNA-binding proteins^3^ that remodel nuclear speckles^4^. The PA-X nuclease, expressed from an alternative PA reading frame^5^, degrades nuclear host transcripts^6^ that organize subnuclear compartments in uninfected cells^7^. How protein and RNA interactions in infected cells influence subnuclear compartments and replication remains poorly understood. Beyond the nucleus, vRNPs are exported by viral proteins M1 (ref. ^8^) and NEP^9^, trafficked via host RAB11A protein^10^ at the reorganized endoplasmic reticulum (ER)^11^, while viral glycoproteins haemagglutinin (HA) and neuraminidase (NA) are synthesized, glycosylated and trafficked through the ER and Golgi^12^ before HA, NA, vRNPs, M1 and the ion channel M2 assemble into budding virions at the plasma membrane.

Systematic native IAV–host PPIs maps are lacking. Existing affinity purification–mass spectrometry (AP–MS) and yeast two-hybrid studies^13–15^ rely on lysed cells, disrupt native cellular architecture, often use non-infected cells and provide no structural information on interaction sites, which remain largely uncharacterized at the atomic resolution for IAV–host complexes.

Here, we applied in-cell cross-linking mass spectrometry (XL-MS)^16^ combined with AlphaFold-based structural modelling and functional analysis to map IAV–host PPIs in infected human lung epithelial cells, yielding residue-to-residue contact sites (within 40 Å, based on the distance constraint of the disuccinimidyl sulfoxide (DSSO) cross-linker used) across hundreds of viral–host pairs.

Integrating XL-MS with structural modelling and functional screens, we uncovered previously unrecognized virus–host interactions and host factors, compartment-specific HA contacts along the ER–Golgi maturation pathway, identified LAT1 as a membrane-associated M2 interactor and revealed that IAV disrupts paraspeckles. We propose that NP and NS1 interactions, PA-X-mediated NEAT1_2 degradation and RNA polymerase II (Pol II) inhibition drive this disassembly. Together, our findings provide a spatially resolved snapshot of IAV–host contact sites and reveal mechanisms of host subversion.

## Results

### Mapping IAV–human contact sites using in-cell cross-linking

**Fig. 1:**
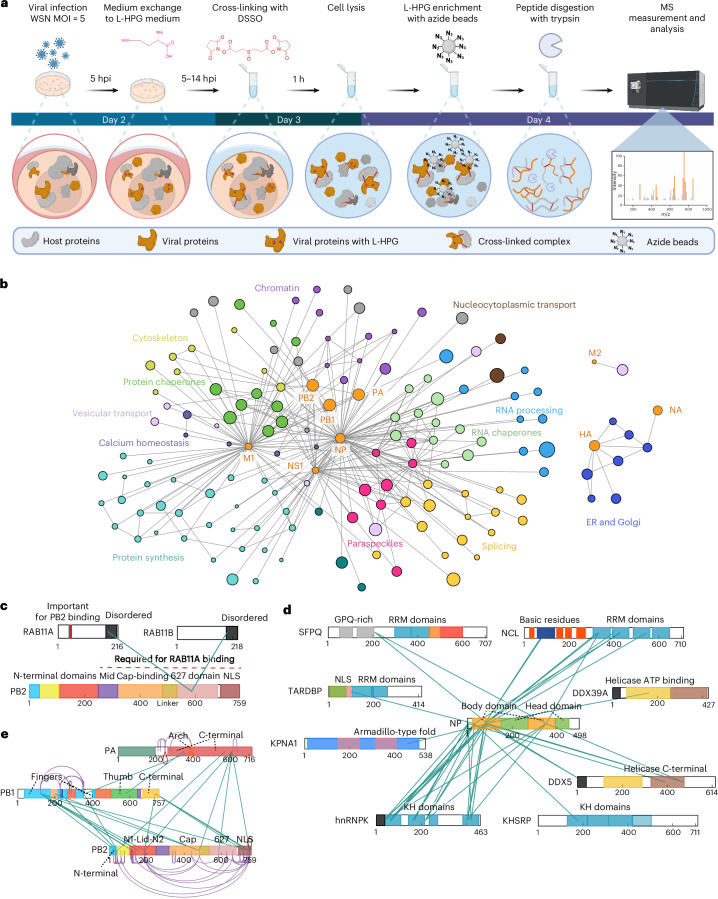
In-cell cross-linking during IAV infection. **a**, Schematic of in-cell cross-linking using SHVIP. IAV-infected cells are labelled with HPG from 5 hpi to 14 hpi, followed by DSSO cross-linking. After quenching, L-HPG-incorporated proteins are enriched via click chemistry, digested with trypsin and cross-linked peptides are analysed by MS. **b**, Virus-centric cross-linking network at a 2% FDR threshold. Viral proteins are shown in orange, and host proteins are coloured by their functional category. Only host proteins linked to viral proteins are shown with names omitted for clarity. The width of each circle is proportional to the protein sequence length. The network including host names is shown in Extended Data Fig. 2. **c**, Interaction between PB2 and RAB11A/B, consistent with the established binding domain of PB2. Interlinks are shown in green, while intralinks are omitted for clarity. **d**, Cross-links between NP and established host factors involved in influenza virus infection. Colour shading represents distinct structural domains, with names shown only for those forming cross-links for clarity. Interlinks are shown in green, while intralinks are omitted for clarity. **e**, Cross-links between subunits of vRdRp. Interlinks are shown in green, while intralinks are in violet. Numbers in **c**–**e** indicate amino acid residue positions in the protein sequence. Diagrams in **a** created in BioRender; Kotova, I. https://biorender.com/uzohrtj (2026). C-terminal, carboxy-terminal; N-terminal, amino-terminal.

To map PPIs and contact sites in cells infected with the A/WSN/33 (H1N1) strain of IAV (hereafter, WSN), we applied structural host–virus interactome profiling (SHVIP)^16^, a recently developed approach that combines in-cell XL-MS with bioorthogonal unnatural amino acid labelling to enrich newly synthesized viral proteins and overcome the sensitivity limits of conventional in-cell XL-MS (Fig. 1a).

**Extended Data Fig. 1:**
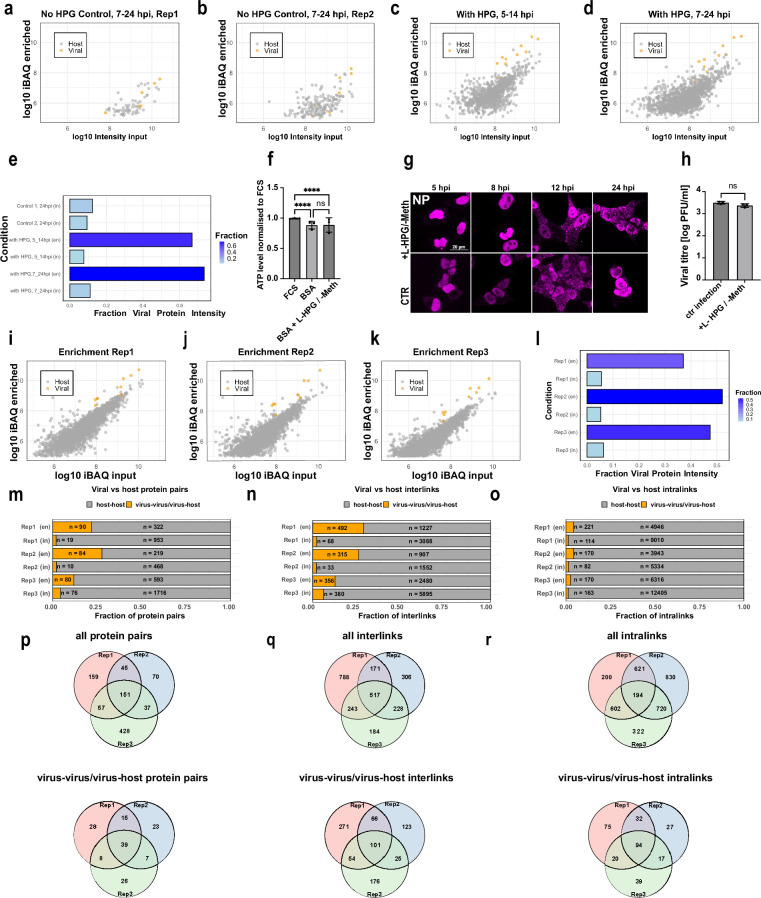
Optimisation of SHVIP and enrichment of IAV proteome in the SHVIP experiment. **a-d**, Determination of optimal time frames for L-HPG pulse labelling of viral proteins. Scatter plots comparing input (x-axis) and enriched (y-axis) log10 iBAQ-values (intensity-based absolute quantification of proteins) of host (grey) and viral (orange) proteins at various time points and conditions. Conditions include no-HPG controls (**a-b**) at 7–24 hpi and HPG-containing samples at 5–14 hpi (**c**) and 7–24 hpi (**d**). **e**, Fraction of viral protein intensities in total proteomic data under various conditions summarizing the data shown in (**a-d**) from enriched (en) and input (in) fractions. **f**, Viability of A549 cells after incubation in three different media conditions: complete media (FCS), infectious media (BSA), and SHVIP media (BSA + HPG / -Meth). Cell viability is normalized to the complete media condition (FCS) (n = 3). Data represent mean ± SD. Statistical significance was determined using an ordinary one-way ANOVA. Significance levels are indicated as follows: **** for p < 0.0001 and ns for not significant. **g**, Maximum projection of A549 cells infected with WSN. The viral nucleoprotein (NP) is used as an infection marker (magenta). Cells were treated with either +HPG/-Meth media or standard infectious media (CTR). Meth stands for methionine. **h**, Viral titres at 14 hpi comparing standard infectious media and +HPG/-Meth media protocols. Data represent mean ± SD (n = 3). Statistical significance was determined using an ordinary one-way ANOVA. Significance levels are indicated as follows: ns for not significant. **i-k**, Scatter plots represent the correlation between input (x-axis) and enriched (y-axis) log10 iBAQ-values of host (grey) and viral (orange) proteins for three independent replicates. **l**, Fraction of viral protein iBAQs in total proteomic data in three replicates summarizing the data shown in i-k, with enriched (en) and input (in) fractions. **m-o**, Fraction of viral-viral/viral-host and host-host protein pairs (**m**), interlinks (**n**), and intralinks (**o**) across three replicates. **p-r**, Venn-diagram depicting overlap among three replicates of protein pairs (**p**), interlinks (**q**) and intralinks (**r**) discriminating between all proteins (top) or only virus-virus/virus-host connections (bottom). Source data

SHVIP labels viral proteins during host shutoff, when viral synthesis dominates^16^. As IAV translation peaks early and declines later^17^, we tested labelling windows and cross-linking times (Extended Data Fig. 1a–e) and chose labelling from 5 hours post-infection (hpi) to 14 hpi with DSSO cross-linking at 14 hpi (Methods and Extended Data Fig. 1c). This captures late-stage vRNP export, cytoplasmic transport and virion assembly^18^ without affecting cell viability or infection progression (Extended Data Fig. 1f–h).

To confirm SHVIP enriches the viral proteome, we compared host and viral protein abundance in enriched versus input samples. Viral proteins were tenfold more abundant across three replicates (Extended Data Fig. 1i–l). XL-MS detected protein pairs and cross-links 2–10× more frequently (Extended Data Fig. 1m–o), with 47% reproducibility across replicates, matching previous studies^19^ and the SHVIP HSV-1 dataset^16^ (Extended Data Fig. 1p–r).

### Extensive in-cell cross-linking network

We analysed raw data from 3 biological SHVIP replicates using a 2% false discovery rate (FDR) threshold at cross-link spectrum match, residue-pair (Supplementary Table 1a) and PPI levels (Supplementary Table 1b). This dataset included 13,588 unique residue-pair cross-links (Supplementary Table 1c) from 2,015 proteins, including 9,613 intraprotein and 3,975 interprotein cross-links. A total of 110 viral–viral and 867 viral–host cross-links were identified, involving 9 viral proteins, 139 host proteins and 198 viral–host protein pairs (Fig. 1b and Supplementary Table 1d), plus 2,626 interprotein cross-links for 914 human protein pairs (Supplementary Table 1c).

**Extended Data Fig. 2:**
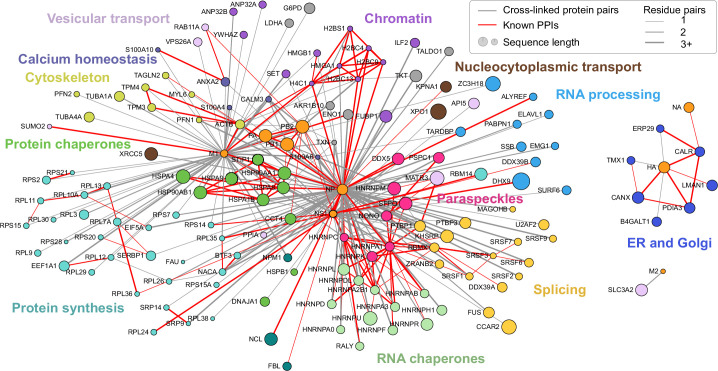
Cross-linking network derived at an FDR threshold of 2% at both residue- and protein-pair levels with additional information mapped. Host proteins not linked to viral proteins are omitted for clarity. Viral proteins are shown in orange, and host proteins are coloured according to their functional category, as in Fig. 1. The width of each circle is proportional to the protein sequence length. Known PPIs are indicated by red lines (curated from the literature for virus-host interactions and retrieved from BioGRID physical interactions for host-host PPIs). The width of the lines corresponds to the number of cross-links for each linked protein pair.

At least 50 viral–host pairs involved host proteins known to bind viral proteins (Supplementary Table 2 and Extended Data Fig. 2). Cross-links also involved host factors known to function in the IAV infection cycle, including RAB11A^18^ (Fig. 1c), KHSRP^20^, TDP-43 (TARDBP)^21^ (Fig. 1d), ANP32A, ANP32B^22^, nucleosomes^23^, microtubules^24^ and chaperonins^25^. Among human pairs, 771 (84%) are BioGRID physical interactions (Supplementary Table 2b), supporting dataset quality.

The interactome is enriched for functional Gene Ontology terms linked to gene expression, RNA processing, splicing, transport, unfolded protein response, protein synthesis, ribosome biogenesis, chromatin organization, calcium homeostasis and phosphorylation (Fig. 1b, Extended Data Fig. 2 and Supplementary Table 3), and localizes to nuclear and cytoplasmic compartments, especially the nucleolus, cytoskeleton and vesicles (Supplementary Table 3), consistent with previous influenza virus PPI studies^13,26^.

**Extended Data Fig. 3:**
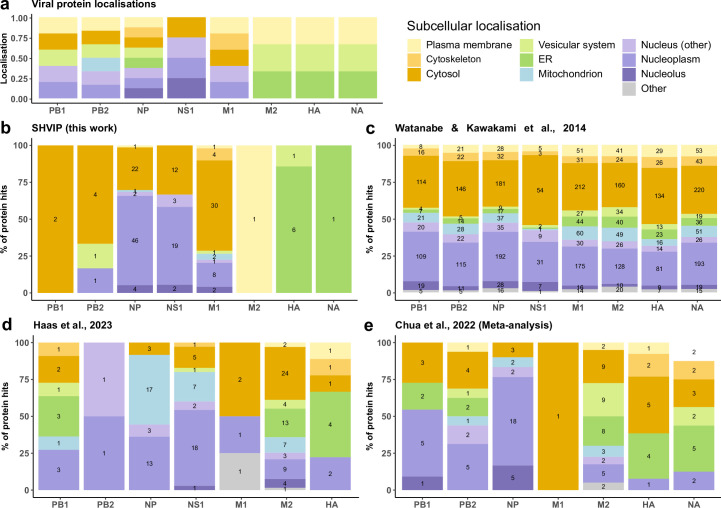
Comparative analysis of subcellular localizations of host interactors of IAV proteins across studies. **a**, Reported subcellular localizations of viral proteins, curated manually from published literature (Supplementary References) and databases (UniProt). **b**, SHVIP (this work): In-cell cross-linking during IAV infection captures endogenous host–viral interactions with spatial resolution. **c**, Watanabe & Kawakami et al., 2014: Individual FLAG-tagged viral proteins of A/WSN/33 (H1N1) were overexpressed in HEK293 cells and subjected to anti-FLAG immunoprecipitation followed by mass spectrometry13. **d**, Haas et al.^15^: A large-scale AP-MS study using codon-optimized, 2X-Strep-tagged viral proteins expressed in three cell types (A549, NHBE, THP-1) across pH1N1, H3N2 and H5N1 strains15. **e**, Chua et al.^26^: A meta-analysis combining host interactors reported across AP-MS, Y2H, RNA-IP, and predictive studies26. Host proteins were considered only if reported in at least three datasets. Only viral proteins identified in SHVIP are shown for other datasets. Bar plots show the percentage distribution of host protein hits (potential interactors) by subcellular compartment for each viral protein. Protein hit counts (n) are overlaid on each bar segment. The subcellular localization annotations are provided in the source data accompanying this figure. Source data

Compared with previous IAV PPI studies, SHVIP showed a more specific localization pattern for viral proteins (Extended Data Fig. 3a) and cross-linked host proteins (Extended Data Fig. 3b) than reported by ref. ^13^ (Extended Data Fig. 3c), ref. ^15^ (Extended Data Fig. 3d) and ref. ^26^ (Extended Data Fig. 3e), with host proteins localizing to compartments matching the viral proteins. The earlier PPI datasets often report incompatible localizations, including widespread mitochondrial interactors for nearly all viral proteins, despite clear evidence for PB2 only (ref. ^27^), and numerous nuclear interactors for ER- and plasma-membrane-associated proteins such as M2, HA and NA. Such inconsistencies may reflect infection-induced localization changes or non-physiological interactions from overexpression and biochemical purification. In contrast, our in-cell XL-MS approach directly captures infection-induced proximities.

**Extended Data Fig. 4:**
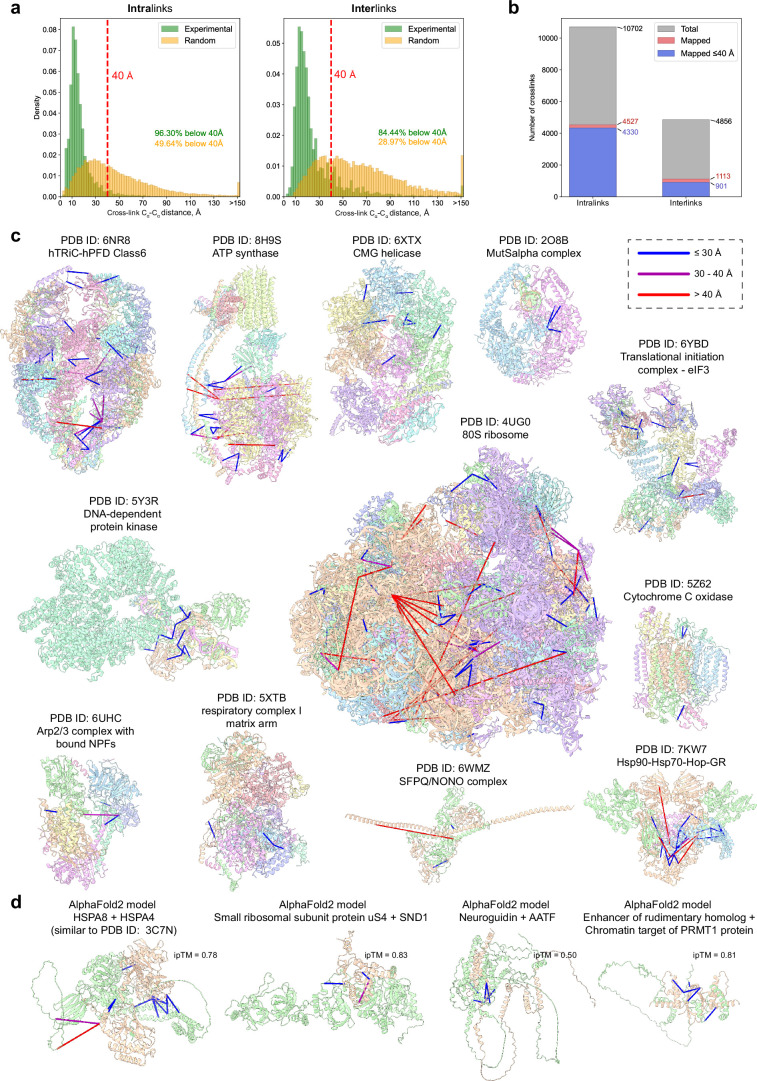
Validation of XL-MS data by available 3D structures and AlphaFold models of cross-linked host PPIs. **a**, Distance histogram of cross-link lengths mapped to 3D structures and AlphaFold. Random distribution corresponds to a random sample of all lysine-residue pairs. The red dashed line indicates the 40 Å threshold. **b**, Bar diagram showing the total number of inter- and intra-cross-links (gray), how many of them could be successfully mapped to PDB structures (red) and how many satisfy the Cα -Cα distance threshold. **c**, Satisfied cross-links (Cα-Cα distance lower than 30 Å) are coloured blue, cross-links in the range from 30 Å to 40 Å are coloured magenta, while cross-links longer than that are coloured red. **d**, Example AlphaFold2-multimer models with cross-links mapped and coloured as in (**c**).

**Extended Data Fig. 5:**
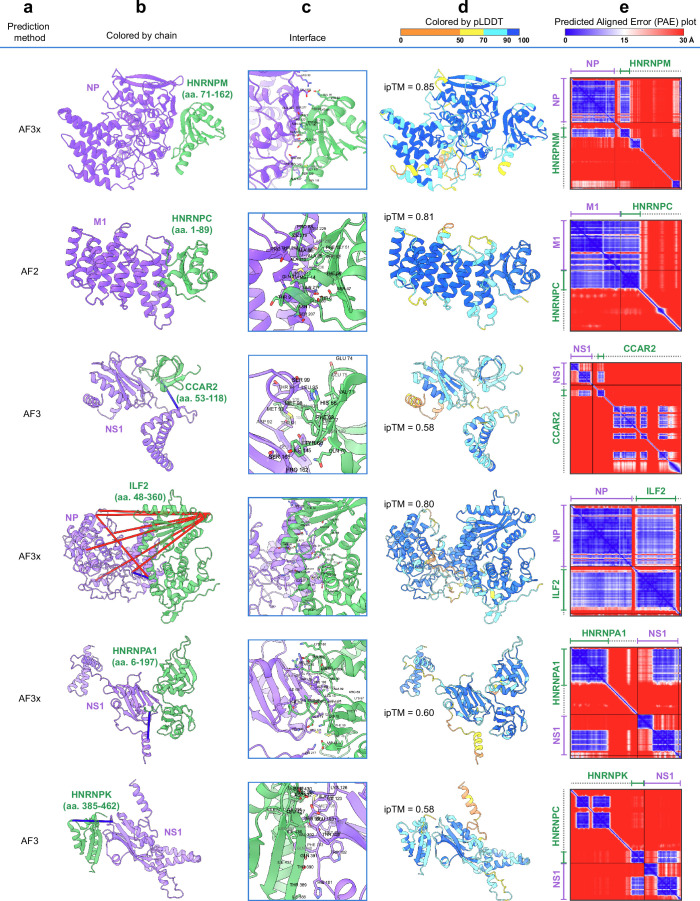
Structural models of IAV proteins in complex with human proteins. Each row presents the structural model of one of the following protein pairs: NP - HNRNPM, M1 - HNRNPC, NS1 - CCAR2, NP - ILF2, NS1 - HNRNPA1, and NS1 - HNRNPK. **a**, The method used for model generation: AlphaFold2-Multimer (AF2), AlphaFold3 (AF3), and AF3x. **b**, Models, where the purple chain corresponds to the IAV protein and the green chain to the human protein. For human proteins, only fragments that confidently interact with the pathogen protein are included. The range of visible amino acids (aa.) is annotated as the first to the last residue shown. Identified interchain cross-links, if present for the shown protein fragments, are marked with lines. Cross-links satisfying the Cα-Cα distance threshold ( < 30 Å) are coloured blue, while longer cross-links are coloured red. **c**, Close up views on interaction sites, with residues shown that have van der Waals radius intersections greater than 0.4 Å with residues from another chain. **d**, Models coloured according to the predicted local distance difference test (pLDDT), reflecting confidence in the predicted residues (higher values indicate greater confidence). The interface predicted template modelling (ipTM) score is also indicated. **e**, Predicted aligned error (PAE) heat maps, where both axes represent residues from the N-terminus to the C-terminus (left to right and top to bottom). The colour of each matrix position represents the confidence in the spatial arrangement of residues relative to one another, with lower errors (blue) indicating higher confidence. Axes parts corresponding to the model chains are coloured to match the chain colouring in the second column. Residues of the host protein hidden from view in panels b and d are indicated by dashed lines. Source data

XL-MS also provides distance constraints that corroborate protein structures or guide structural modelling^28^. Mapping host–host cross-links onto Protein Data Bank (PDB) structures showed 93% of Cα–Cα distances between cross-linked residues fall within the DSSO constraint of 40 Å (Extended Data Fig. 4). For viral complexes such as RdRp, satisfaction rates are difficult to calculate because of conformational and oligomeric heterogeneity^29^. Nonetheless, the RdRp cross-linking pattern (Fig. 1e) aligns with purified RdRp^29^. No PDB structures captured resolved IAV–human cross-links, underscoring the difficulty of structurally capturing these interactions. Several IAV–human interactions were modelled confidently using AlphaFold2-multimer^30^, AlphaFold 3 (ref. ^31^), and the cross-link-guided protocols AF3x^32^ and GRASP^33^ (Extended Data Fig. 5 and Supplementary Table 4). Many pairs could not be modelled, probably because host–pathogen systems lack the co-evolutionary signals and same-species sequence pairing that AlphaFold relies on^30^.

Overall, the overlap of identified cross-links with functional host protein categories, known host factors, IAV-characteristic PPIs and AlphaFold models, together with consistent subcellular localization, demonstrates that SHVIP delivers a structurally informative IAV–host interactome and captures interactions in their native cellular context.

### In situ validation of selected host–IAV interactions by proximity ligation assay

**Fig. 2:**
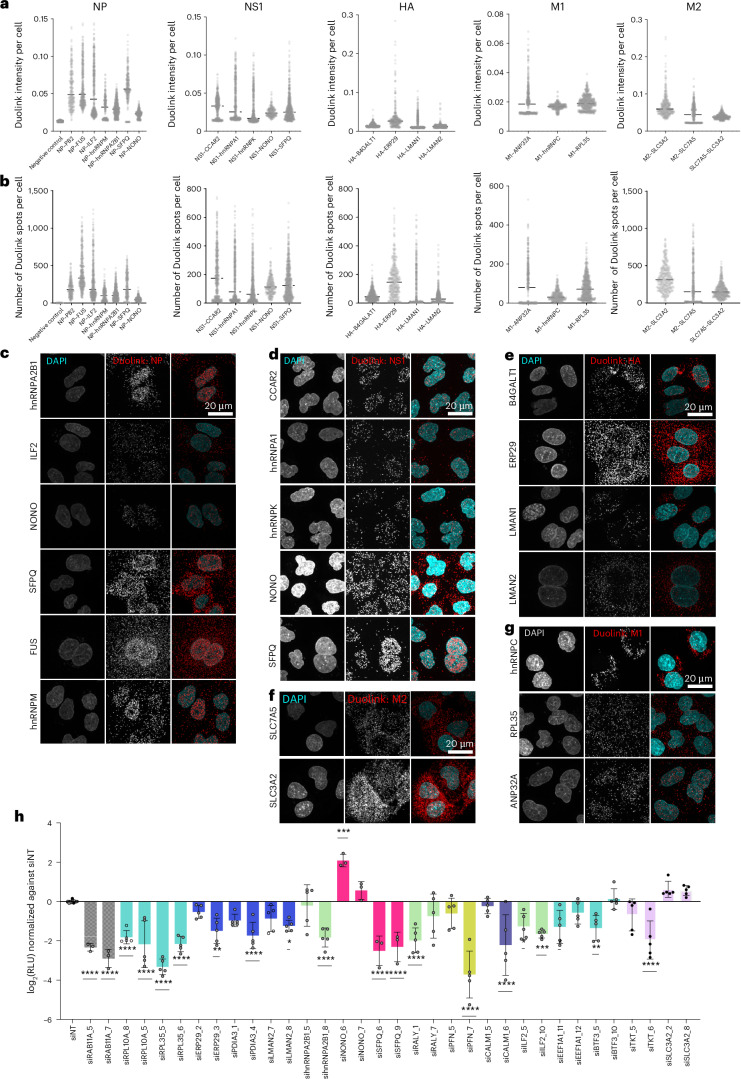
In situ validation of selected viral–host proximities by PLA and functional loss-of-function screening. **a**,**b**, PLA quantification for selected viral–host protein pairs in IAV-infected cells at 14 hpi. PLA signal is shown as total PLA intensity per cell (**a**) and number of PLA puncta per cell (**b**) for NP, NS1, HA, M1 and M2. Each dot represents one cell; cells from at least two independent experiments were pooled for visualization. The horizontal lines indicate the median. The same negative control condition was used for all PLA measurements. **c**–**g**, Representative PLA images for selected viral–host protein pairs: NP (**c**), NS1 (**d**), HA (**e**), M2 (**f**) and M1 (**g**). For each condition, DAPI (nuclei), PLA signal and merged images are shown. Representative images from at least two independent experiments with similar results are shown. Scale bars, 20 µm. See also Supplementary Fig. 1. **h**, log_2_-transformed relative luciferase units (log_2_ (RLU)) normalized to a non-targeting siRNA control (siNT). Bars are coloured by functional category of the host target as in Fig. 1b; RAB11A siRNAs (positive control) are shown in patterned grey. *X*-axis labels indicate the host gene targeted (format: si(gene)_(siRNA index)). Data are represented as mean ± s.d. from *n* = 3–5 (depending on the sample, see Source data) independent biological replicates (independent infections; unit of study). *P < 0.05, **P < 0.01, ***P < 0.001, ****P < 0.0001 (one-way analysis of variance (ANOVA) and Dunnett’s multiple comparisons test; reference, siNT, two-sided). Exact *P* values are provided in the Source data. See also Extended Data Fig. 6. Source data

We validated viral–host interactions in IAV-infected cells by proximity ligation assay (PLA; Duolink) at 14 hpi on cross-linked protein pairs, selected from those for which reliable immunofluorescence-grade antibodies from different species were available. Signal intensity and puncta per cell were quantified (Fig. 2a–g and Supplementary Fig. 1).

We included established positive controls (NP–PB2, SFPQ–NONO and SLC3A2–SLC7A5) and single-antibody controls for each antibody (Supplementary Fig. 1a–c). Positive controls consistently produced robust PLA signals with high puncta numbers, defining the dynamic range of detectable in-cell proximity, whereas single-antibody conditions yielded minimal background. PLA signal strength does not necessarily correlate with interaction affinity: M2–SLC3A2 and M2–SLC7A5 showed stronger signals than the SLC3A2–SLC7A5 control. This probably reflects proximity, protein abundance, subcellular localization and antibody performance. Using these benchmarks, all viral–host pairs showed reproducible PLA signals above single-antibody background and negative controls, ranging from weak (for example, M1–ANP32A and HA–LMAN1/2) to strong (for example, NP–FUS, NP–ILF2, NP–SFPQ, HA–ERP29 and M2–SLC3A2).

### Cross-linking network and short interfering RNA screening reveal host factors and functional hypotheses

**Extended Data Fig. 6:**
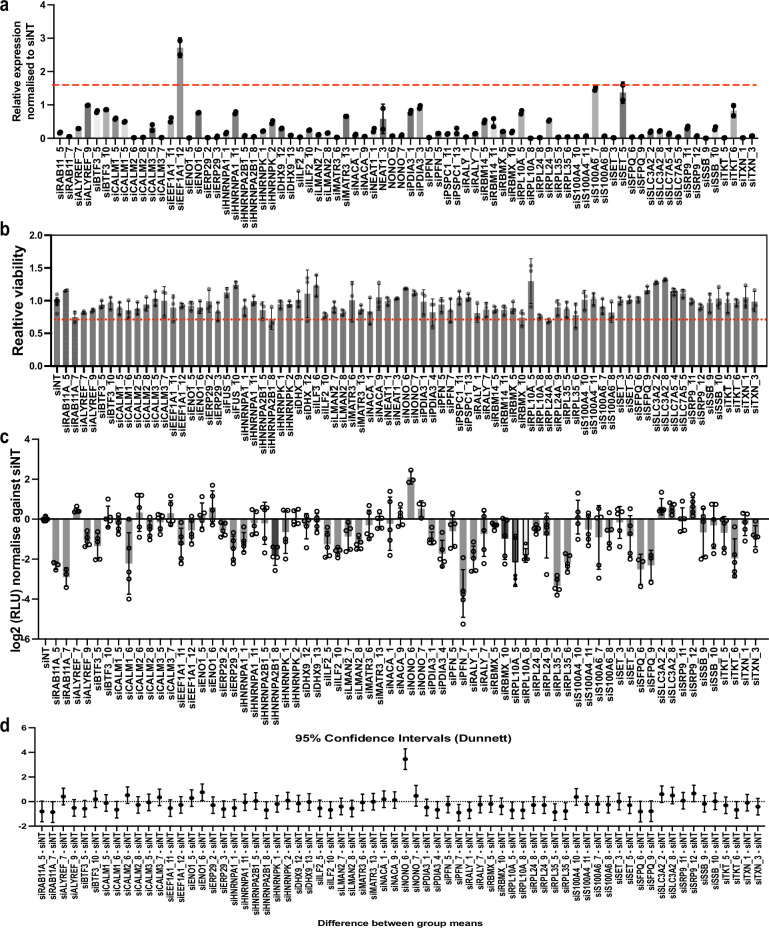
The results of the siRNA screen. **a**, Knockdown efficiency of target genes in A549 cells using siRNA, assessed by qPCR. Data are presented as mean ± SD from n = 3 independent biological replicates (independent experiments; unit of study), normalized to the non-targeting control (siNT). The dashed red line indicates the value obtained for the control. **b**, Viability was measured using the CellTiter-Glo assay and normalized to the non-targeting control. Data are presented as mean ± SD from n = 3 independent biological replicates (independent experiments; unit of study). The red dashed line indicates a 70% viability cut-off; siRNAs below this threshold were excluded from further analysis. **c**, Luciferase assay in A549 cells with target gene knockdown, infected with recombinant WSN (MOI 0.01, PB2-T2A-NanoLuc) for 48 h. Data are normalized to siNT and presented as mean ± SD from n = 4 independent biological replicates (independent experiments; unit of study). Technical replicates were averaged prior to analysis. Statistical significance was determined using ordinary one-way ANOVA with Dunnett’s multiple comparisons test, two-sided, comparing each condition to siNT. **d**, Confidence interval (CI) plot for luciferase activity showing comparisons between knockdowns and siNT. Data are presented as mean ± 95% CI from n = 4 independent biological replicates (independent experiments; unit of study). Statistical analysis was performed using ordinary one-way ANOVA with Dunnett’s multiple comparisons test, two-sided, comparing each condition to siNT. Source data

To test whether our cross-linking network identifies host factors in IAV infection and generates functional hypotheses, we performed a short interfering RNA (siRNA) loss-of-function screen targeting selected cross-linked host proteins (Fig. 2h and Extended Data Fig. 6). We prioritized proteins with at least three cross-links to viral proteins, excluded well-studied host factors and included six with fewer cross-links—*ERP29*, *LMAN2*, *EEF1A1*, *SET*, *SSB* and *RALY*—based on roles in nucleic acid metabolism, gene expression regulation and ER localization. We also included *SLC3A2*, the sole M2 interactor, and *RAB11A* as a positive control. Infection efficiency was quantified by luciferase assay^34^ (Fig. 2h and Extended Data Fig. 6c,d). *RAB11A* knockdown reduced replication approximately tenfold (Fig. 2h), consistent with previous reports^18^ and validating the assay. Of the 33 genes tested, 15 significantly affected luciferase activity. *RPL35* and *RPL10A* knockdown reduced replication, whereas other ribosomal proteins had no effect, suggesting specific functions (Fig. 2h and Extended Data Fig. 6c,d) consistent with NS1 regulating translation^35^.

Knockdown of *SLC3A2*, which cross-linked to M2, increased infection but was significant for only one of two siRNAs (Supplementary Fig. 2a–c). At a less stringent threshold, we detected a second M2 cross-link to SLC7A5, which dimerizes with SLC3A2 to form LAT1 (ref. ^36^) (Supplementary Fig. 2a). Functionally, siRNA perturbation of LAT1 components mildly increased viral replication in reporter and titration assays; this was significant for *SLC7A5*, whereas *SLC3A2* showed a similar non-significant trend (Supplementary Fig. 2e,f). Colocalization (Supplementary Fig. 2g–o) and PLA (Fig. 2a,b,f) further supported the interaction. These data indicate that LAT1 can restrict IAV infection, but with limited, context-dependent contribution (Supplementary Note 1).

The NP–hnRNPM interaction, for which we obtained a high-confidence AlphaFold model (Extended Data Fig. 5), was independently confirmed during review of this article by a focused study identifying hnRNPM as a vRNP-interacting host factor in influenza virus infection^37^.

These findings establish SHVIP as a robust approach for identifying host proteins critical for IAV infection, including several linked to ER function and paraspeckles, which are discussed later.

### The steps of the early secretory pathway of HA mapped by XL-MS

**Fig. 3:**
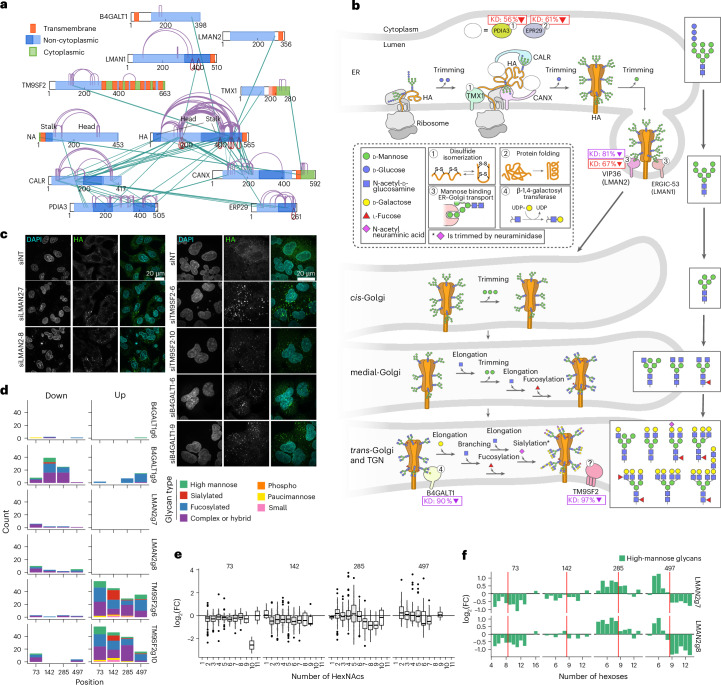
Analysis of interactions at membrane compartments. **a**, HA cross-linking network with sequence bar diagrams with structural domains, coloured by orientation across the ER membrane. Numbers indicate amino acid residue positions in the protein sequence. **b**, Early secretory pathway of HA. Per cent values indicate the effect of the corresponding gene knockdown (KD) on IAV production in luciferase (red) or multicycle titration (magenta) assays at 48 hpi. Insets show presumed functions or maturation steps; the question mark next to TM9SF2 denotes an uncharacterized mechanism. **c**, HA localization in A549 cells after siRNA-mediated knockdown and WSN infection (MOI 3, 14 hpi). Two independent siRNAs were used per gene; representative images from at least two independent experiments are shown. Scale bars, 20 µm. **d**, Distribution of glycan types detected on HA across individual N-linked glycosylation sites after knockdown. gN labels identify individual siRNAs targeting the indicated gene. **e**, HA glycan maturation after *B4GALT1* knockdown (average of both siRNAs). log_2_-transformed fold changes (FC) of TMT-based HA glycopeptide intensities are plotted against N-acetylhexosamine (HexNAc) count for individual glycosylation sites, using the thresholds in Extended Data Fig. 7c. Box plots show the median, interquartile range, 1.5× interquartile range whiskers and outliers; points are individual glycopeptides. *N* = 3 biological replicates (unit of study, glycopeptide). **f**, High-mannose glycan profiles at HA glycosylation sites after *LMAN2* knockdown. Bars show the mean limma-derived log_2_-transformed fold change versus siNT across all HA glycopeptides at each hexose number, from *n* = 3 independent biological replicates (unit of study, glycopeptide). Red lines mark the approximate transition from less-trimmed ER-associated species (right) to more-trimmed Golgi-processed species (left). TGN, *trans*-Golgi network. Source data

We found HA cross-linked to ER and Golgi proteins, with all cross-links supporting the same membrane orientations (Fig. 3a). HA is synthesized in the ER and undergoes N-linked glycosylation, folding and processing in the ER and Golgi before export to the plasma membrane^38^. These cross-links indicated XL-MS can reconstruct sequential steps of the HA secretory pathway across these compartments (Fig. 3b).

**Extended Data Fig. 7:**
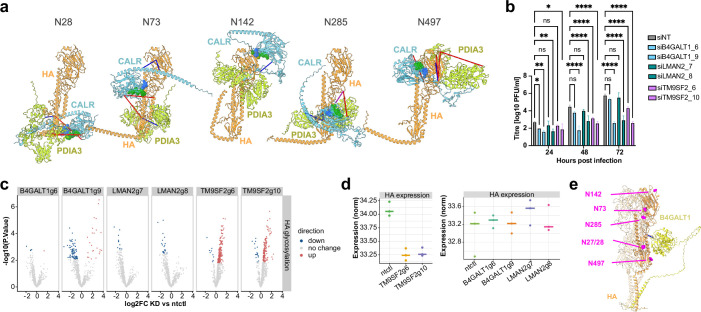
Structural and functional analysis of host factors involved in HA glycosylation. **a**, AlphaFold3 models of glycosylated HA monomers bound to CALR and PDIA3, with cross-links mapped. Each model represents a distinct glycosylation site; glycans are shown as spheres (N-Acetylglucosamine, blue; Mannose, green). Cross-links are coloured by Cα-Cα distance: ≤30 Å, blue; 30–40 Å, magenta; >40 Å, red. **b**, IAV growth kinetics in A549 cells transfected with siRNA against the indicated genes or non-targeting siRNA (siNT) for 72 h before infection with WSN (MOI 0.001). Data are mean ± SD from n = 3 independent biological replicates. **c**, Volcano plots of HA glycopeptide changes for each knockdown versus siNT control (ntctl). x axis, log2 fold change of TMT-based intensities (limma linear model, moderated two-sided t-test with empirical Bayes); y axis, −log10 unadjusted P value. Coloured points (red, up; blue, down) pass both thresholds: Benjamini–Hochberg-adjusted P < 0.05 and |log2 fold change | > log2(1.2). **d**, HA abundance across knockdowns, as VSN-normalized log2 TMT intensities from the full-proteome dataset. Dots represent biological replicates (n = 3); bars indicate medians. **e**, AF3x modelling of the HA trimer with B4GALT1 did not define a confident interface but indicated proximity of B4GALT1 to affected N-linked glycosylation sites. The cross-link is shown in blue. gN labels in panels c and d identify individual siRNAs targeting the indicated gene. Source data

The earliest captured step is defined by HA cross-links to calnexin (CANX) and calreticulin (CALR), key chaperones in glycoprotein folding and trafficking^39^, consistent with roles in HA processing^40^. These cross-links localized to HA stalk and head domains, aligning with AlphaFold 3 models of CALR bound to N-glycans on HA (Extended Data Fig. 7a). HA also cross-linked with PDIA3 (ERP57), supporting its role in HA disulfide bond formation^41^, and with another disulfide isomerase TMX1 and the ER chaperone ERP29 (ref. ^42^), neither of which have been previously linked to HA maturation. Knockdown of *PDIA3* and *ERP29* reduced IAV infection (Fig. 2h).

Further along the pathway, HA cross-linked with LMAN1 and LMAN2 (ERGIC-53 and VIP36; LMAN2 only in the non-stringently filtered list, Supplementary Table 1e–g), lectins involved in glycoprotein trafficking^43,44^. Consistent with the role in HA maturation, *LMAN2* knockdown significantly reduced IAV infection (Fig. 2h). Although LMAN1 supports glycoprotein production in various viruses^43^, LMAN2 has not been implicated in IAV infection. HA also cross-linked with B4GALT1, suggesting further HA processing, and with TM9SF2, which is implicated in Golgi and endosomal sorting^45,46^ and whose overexpression inhibits IAV infection^47^.

Knockdown of *LMAN2*, *B4GALT1* or *TM9SF2* reduced multicycle WSN replication in A549 cells (5 of 6 siRNAs; Extended Data Fig. 7b). *TM9SF2* knockdown showed the strongest phenotype, with reduced titres at all time points through 72 hpi, indicating a sustained defect in viral spread. To define roles in HA maturation, we stained HA upon knockdown of *LMAN1*, *LMAN2*, *ERP29*, *PDIA3*, *B4GALT1* and *TM9SF2*. HA remained diffuse after *LMAN1*, *PDIA3* and *ERP29* knockdown (Supplementary Fig. 3a), whereas *LMAN2*, *B4GALT1* and *TM9SF2* knockdown increased punctate HA accumulation (Fig. 3c), suggesting impaired ER–Golgi trafficking or maturation.

We therefore applied deep quantitative glycoprofiling (DQGlyco)^48^ to measure HA glycosylation changes (Supplementary Fig. 3b). The knockdowns did not affect cell viability and efficiently depleted the corresponding proteins (Extended Data Fig. 6b and Supplementary Fig. 3b,c). DQGlyco detected N-glycosylation at four HA sites (N73, N142, N285 and N497); N27/28 was not detected due to the tryptic digest (Methods). In control cells, HA showed substantial site-specific glycosylation heterogeneity, with multiple glycan types detected across different sites and within sites, reflecting progressive glycan maturation and divergent processing outcomes along the secretory pathway.

Across knockdowns, HA glycosylation was consistently altered (Extended Data Fig. 7c) amid broader proteome and glycosylation perturbations (Supplementary Fig. 3d,e). *TM9SF2* knockdown, which most strongly reduced infection (Extended Data Fig. 7b), also had the strongest effects on HA maturation, reducing HA abundance (Extended Data Fig. 7d) and relatively enriching glycosylated HA species (Extended Data Fig. 7c). HA glycosylation increased across all sites and glycan types (Fig. 3d and Supplementary Fig. 3f).

*B4GALT1* depletion reduced overall HA glycosylation, most strongly with the more efficient knockdown (siRNA g10; Extended Data Fig. 7c). As B4GALT1 catalyses galactose addition late in complex N-glycan maturation, its depletion should impair further branching and extension (Fig. 3b), causing selective loss of highly processed, high N-acetylhexosamine species. Site-resolved analysis confirmed reduced high N-acetylhexosamine-containing glycans with counts greater than six at N285 and N497 (Fig. 3e and Supplementary Fig. 3h), whereas N142 and N73 showed a more uniform reduction. This matches the XL-MS-guided structural model (Extended Data Fig. 7e), in which N285 and N497 are close to B4GALT1, whereas N142 and N73 are more distant.

*LMAN2* knockdown decreased all major glycan types (Fig. 3d). At N285 and N497, *cis*-Golgi-specific glycans increased, whereas ER-specific high-mannose glycans decreased only at N497 (Fig. 3f and Supplementary Fig. 3i). This pattern contradicts a simple ER-retention model, which predicts accumulation of ER-type high-mannose glycans when ER-to-Golgi transport is impaired. One possible explanation would be compensatory activity of the related lectin LMAN1, which could enhance ER-to-Golgi transport. However, LMAN1 protein levels remained unchanged upon LMAN2 depletion (Supplementary Fig. 3g). Together, these observations suggest that LMAN2 influences ER quality control, ERGIC/*cis*-Golgi transit of HA and site-specific glycan processing, rather than serving as a simple ER-export factor.

These results outline a pathway in which HA interacts with CANX, CALR, PDIA3, ERP29 and TMX1 in the ER; traffics via LMAN1 and LMAN2; and is processed by B4GALT1 in the Golgi under TM9SF2 regulation (Fig. 3b). DQGlyco indicates site-specific effects on HA glycosylation for tested host factors, except TM9SF2, which affects all sites and glycan types. Thus, in-cell XL-MS effectively maps infection pathways in native cells.

### IAV proteins interact with paraspeckles and disrupt their integrity

**Fig. 4:**
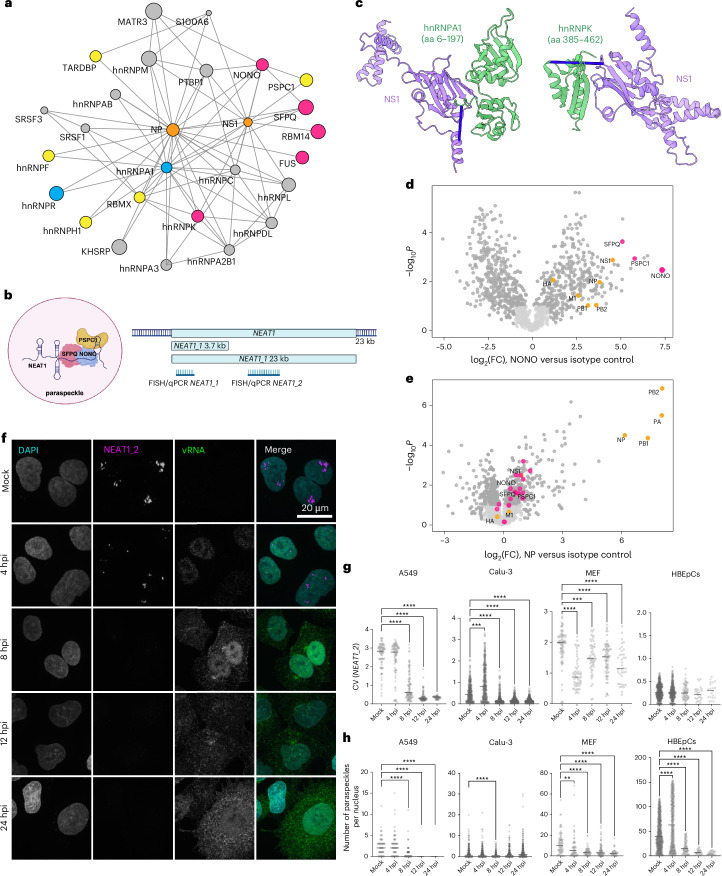
Impact of IAV infection on paraspeckle formation and *NEAT1* expression. **a**, Cross-linking network between paraspeckle and viral proteins. Pink indicates the protein is essential for paraspeckle formation; blue, important; yellow, localized to paraspeckles but dispensable; grey, other proteins cross-linked to both paraspeckle; and orange, viral proteins. **b**, Paraspeckle structure, showing the position of FISH probes and qPCR primers used in this study on NEAT1 sequence (right). **c**, AlphaFold models of NS1 and paraspeckle proteins hnRNPK and hnRNPA1 (Extended Data Fig. 5). Cross-links are indicated with blue sticks. **d**,**e**, Volcano plots of AP–MS of NONO (**d**) or NP (**e**) versus IgG controls from WSN-infected A549 cells (14 hpi), showing fold change (log_2_) of protein LFQ values versus significance (−log_10_*P*, 2-sided Student’s *t* test without multiple hypothesis correction). Data are derived from *n* = 3 independent biological replicates (independent infections), each corresponding to one AP–MS experiment (unit of study). **d**, NONO and its known interactors are coloured pink; viral proteins are orange. **e**, Proteins that were also identified in SHVIP are highlighted; proteins coloured as in **d**. **f**, Maximum projection of confocal microscopy images of A549 cells infected with WSN (MOI 3) at 4 hpi, 8 hpi and 12 hpi. NEAT1, magenta; vRNA (PB2 fragment), green; and DNA (DAPI), grey. Representative images from at least two independent experiments with similar results are shown. **g**,**h**, CV of NEAT1_2 (NEAT1_1 in MEFs) in the nucleus across different cell lines infected with WSN (**g**) and the number of paraspeckles per nucleus across different cell lines infected with WSN (**h**). Each point represents a single nucleus; quantification from 1 representative experiment with *n* = 20–526 nuclei per condition (unit of measurement: nucleus). All experiments were independently repeated at least twice with similar results. Statistical analysis was performed using ordinary one-way ANOVA with Dunnett’s multiple comparisons test, two-sided. *P < 0.05, **P < 0.01, ***P < 0.001, ****P < 0.0001. Exact *P* values are provided in the Source data. Diagram in **b** created in BioRender; Kotova, I. https://biorender.com/b92y974 (2026). Source data

**Extended Data Fig. 8:**
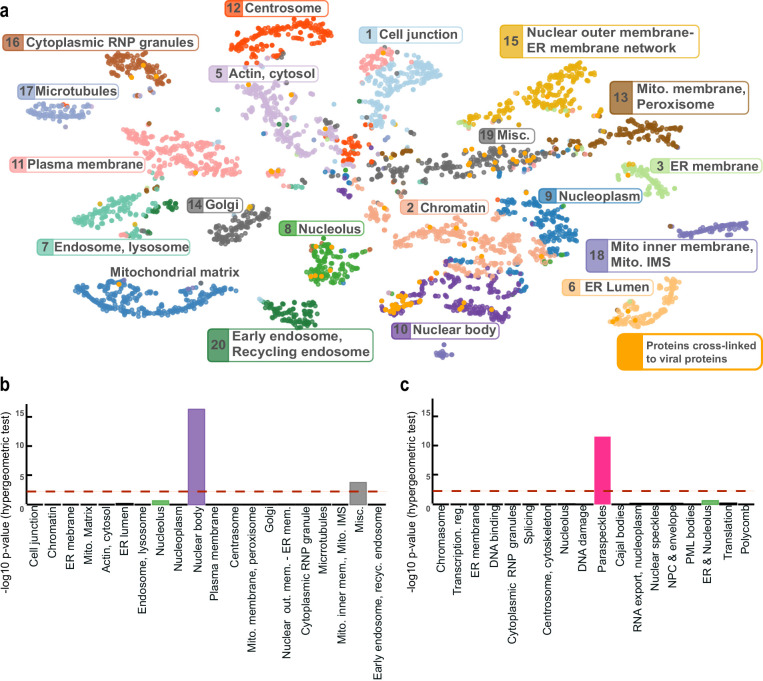
Spatial proteomic context of IAV–host protein interactions. **a**, Mapping of host proteins cross-linked to IAV proteins onto the proximity-dependent biotinylation map of the human proteome49. Each coloured cluster represents a spatially resolved cellular compartment. Crosslinked host proteins were overlaid onto the map to infer their spatial context. **b**, Enrichment analysis of IAV-cross-linked host proteins using the Go et al. proximity labelling organelle map49. Significant enrichment was observed in the nuclear bodies (purple bar). **c**, Spatial enrichment using the updated nuclear body proteome dataset, revealing strong enrichment in paraspeckles. Enrichment was assessed using a one-sided hypergeometric test based on the overlap between IAV cross-linked host proteins and proteins assigned to each subcellular category. P values are unadjusted. Subcellular localization annotations are provided in the source data accompanying this figure. Source data

To determine the subcellular localization of IAV–host protein interactions, we mapped host proteins cross-linked to viral proteins onto reference spatial proteomics datasets, including a global proximity-dependent biotinylation map of human cellular compartments^49^ (Extended Data Fig. 8a). Enrichment analysis showed a striking overrepresentation of IAV-associated host proteins in nuclear bodies (Extended Data Fig. 8b). A high-resolution nuclear body proteome^50^ further enabled us to refine this localization, identifying paraspeckles as a highly enriched subnuclear structure where IAV–host interactions are concentrated (Extended Data Fig. 8c). SHVIP identified cross-links from NP and NS1 to 14 paraspeckle proteins (Fig. 4a). Paraspeckles are nuclear membraneless organelles built around long non-coding RNA NEAT1_2, essential for structural integrity (Fig. 4b)^51^. Three proteins—NONO, SFPQ and PSPC1—form the paraspeckle core. Additional proteins such as FUS, CPSF6 and hnRNPK contribute to paraspeckle structure and function^52–54^. Paraspeckles modulate gene expression and may influence viral replication and pathogenesis through interactions with viral components^55,56^.

Supporting our SHVIP data (Fig. 4a), prior studies linked paraspeckle proteins SFPQ, hnRNPK, hnRNPA1 and RBM14 to the IAV infection cycle^3,57,58^ through interactions with IAV proteins NP and NS1 (refs. ^59–62^). We also found that knockdown of *SFPQ* and *NONO* affected IAV infection (Fig. 2h). Well-scoring structural models supported NS1 interactions with hnRNPK and hnRNPA1 (Fig. 4c and Extended Data Fig. 5). Reciprocal immunoprecipitations (IPs) confirmed NP interactions with NONO, SFPQ and PSPC1 in HEK293T cells (Supplementary Fig. 4a,b) and WSN-infected A549 cells (Supplementary Fig. 4c). NONO AP–MS showed enrichment of NONO cross-linking partners SFPQ, PSPC1, NS1 and NP (Fig. 4d). NP AP–MS showed higher abundance of NP cross-linked proteins than the isotype control (Fig. 4e and Supplementary Fig. 4e). Together, these data indicate that IAV NP and NS1 interact with paraspeckle proteins, particularly NONO. Moreover, the overlap between NP, NS1 and NONO interactors (Supplementary Fig. 4d,e) suggests that other paraspeckle proteins may also play a role in the viral replication cycle during IAV infection.

To assess IAV effects on paraspeckles, we used fluorescence in situ hybridization (FISH) to localize and quantify NEAT1_1 and NEAT1_2 in WSN-infected versus non-infected A549 cells (Fig. 4f and Supplementary Fig. 4f). Paraspeckles were present in non-infected cells, but their number and NEAT1_2 coefficient of variation (CV), which measures signal heterogeneity independent of the mean, progressively decreased during infection to very low levels between 4 hpi and 8 hpi (Fig. 4g–h and Supplementary Fig. 4f,g). In A549 cells overexpressing mEGFP–NONO and mEGFP–SFPQ, both proteins colocalized with NEAT1_2 (Supplementary Fig. 4h–k) but redistributed to the nucleoplasm during IAV infection. Similar reductions were observed in WSN-infected Calu-3, mouse embryonic fibroblasts (MEFs) and HeLa cells (Supplementary Fig. 4l,m,w,x), contrary to a previous report of increased paraspeckles upon IAV infection in HeLa^57^.

We extended our analysis to additional IAV strains and primary human bronchial epithelial cells (HBEpCs). Paraspeckle numbers and CV were reduced in HBEpCs, although less so, probably because of lower infection efficiency (Supplementary Fig. 4n). H1N1/California/pdm09 (Supplementary Fig. 4o,p) and H3N2/Aichi/68 (Supplementary Fig. 4q,r) similarly reduced NEAT1_2 CV and paraspeckle numbers in A549 cells and primary HBEpCs (Supplementary Fig. 4s–v).

Altogether, the results show that IAV consistently disrupts paraspeckles between 4 hpi and 8 hpi across different cell lines, including primary cells, and across different viral strains.

**Fig. 5:**
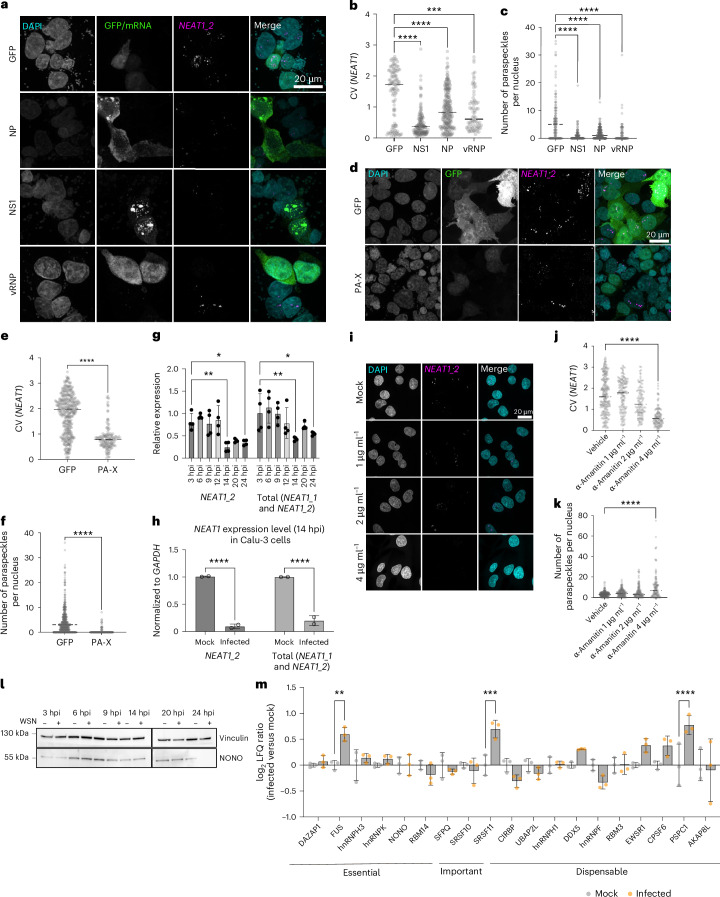
Mechanisms of paraspeckle disruption by IAV proteins. All infections are with WSN at MOI = 3. **a**, Maximum-projection FISH confocal images of HEK293T cells transfected with GFP (control), WSN NP, NS1 or a mini-replicon vRNP system; 24 hours post-transfection. NEAT1_2 (paraspeckles), magenta; GFP or viral mRNAs, green; DAPI, cyan. Representative of at least two experiments. Scale bar, 20 µm. **b**,**c**, CV of NEAT1_2 (**b**) and paraspeckles per nucleus (**c**) across conditions in GFP-positive cells; each point indicates 1 nucleus; representative experiment, *n* = 72–194 nuclei per condition; repeated at least 2× with similar results. Ordinary one-way ANOVA with Dunnett’s multiple comparisons test, two-sided. **d**, Maximum-projection confocal images of HEK293T cells transfected with GFP or cotransfected with PA-X and GFP, 24 hours post-transfection. NEAT1_2, magenta; GFP, green; DAPI, grey. Representative of at least two experiments. **e**,**f**, Nuclear NEAT1_2 CV (**e**) and paraspeckles per nucleus (**f**) in GFP-positive cells; each point indicates 1 nucleus; representative experiment, *n* = 134–399 nuclei per condition; repeated at least 2× with similar results. Ordinary one-way ANOVA with Dunnett’s multiple comparisons test, two-sided. **g**, qPCR of NEAT1_2 and NEAT1_1 in infected A549 cells, normalized to *GAPDH* and mock at each corresponding time point; mean ± s.d., *n* = 4 independent biological replicates. **h**, qPCR of NEAT1_2 and NEAT1_1 in infected Calu-3 cells, normalized to *GAPDH* and mock-infected cells; mean ± s.d., *n* = 2 biological replicates. **i**, Maximum-projection confocal images of NEAT1_2 in A549 cells treated with α-amanitin for 20 hours. NEAT1_2, magenta; DAPI, cyan. Representative of at least two experiments. Scale bar, 20 μm. **j**,**k**, NEAT1_2 signal CV (**j**) and paraspeckles per nucleus (**k**); each point indicates 1 nucleus; representative experiment, *n* = 100–207 nuclei per condition; repeated at least 2×. Ordinary one-way ANOVA with Dunnett’s multiple comparisons test, two-sided. **l**, Western blot of NONO in infected A549 cells at 3–24 hpi; vinculin, loading control. Representative of at least two experiments. **m**, log_2_ LFQ ratio of paraspeckle proteins in infected A549 cells at 14 hpi (*n* = 3 biological replicates). Data are mean ± s.d. For **g**, **h** and **m**, ordinary one-way ANOVA, two-sided. *P < 0.05, **P < 0.01, ***P < 0.001, ****P < 0.0001. Source data

To determine which viral proteins disrupt paraspeckles, we expressed NP and NS1 in HEK293T cells. Both proteins reduced paraspeckle abundance (Fig. 5a–c), whereas M2, HA and NA overexpression did not affect paraspeckle number (Supplementary Fig. 4y–z). Co-expression of vRNPs further intensified paraspeckle dissolution compared with NP alone (Fig. 5a–c), suggesting that additional vRNP components affect paraspeckle integrity. We hypothesized that the viral endonuclease PA-X may contribute, as it is produced via a frameshift^6^ from PA mRNA and its cleavage motif GCUG^6^ occurs 139 times in NEAT1_2. Indeed, PA-X overexpression significantly reduced paraspeckle number (Fig. 5d–f). *NEAT1_2* levels were reduced by 50% at 12 hpi in A549 cells and by 15% at 14 hpi in Calu-3 cells (Fig. 5g,h), whereas paraspeckle disruption occurred earlier (4–8 hpi) (Fig. 4g,h and Supplementary Fig. 4l), suggesting that structural disassembly precedes NEAT1 downregulation.

As paraspeckles depend on active transcription^52^ and viral polymerase binds and inhibits Pol II^63^, we tested whether Pol II inhibition disrupts paraspeckles. In non-infected A549 cells, the Pol II inhibitor α-amanitin^64^ disrupted paraspeckles in a concentration-dependent manner from 20 hours post-treatment (Fig. 5i–k). As disruption begins within 8 hours of infection, this delay suggests Pol II inhibition contributes at later stages. NONO levels remained stable in mock and infected A549 cells, indicating that disassembly is not caused by NONO downregulation (Fig. 5l). Other essential proteins, including SFPQ and hnRNPK, were also unchanged, whereas FUS, SRSF10 and PSPC1 increased upon infection (Fig. 5m).

These findings suggest a three-pronged IAV attack on paraspeckle formation: NS1- and NP-mediated interactions with paraspeckle protein components, PA-X-dependent degradation of architectural NEAT1_2 RNA, and possible Pol II-mediated NEAT1_2 transcriptional downregulation.

### Disruption of paraspeckles enhances IAV replication by liberating proviral host factors

**Fig. 6:**
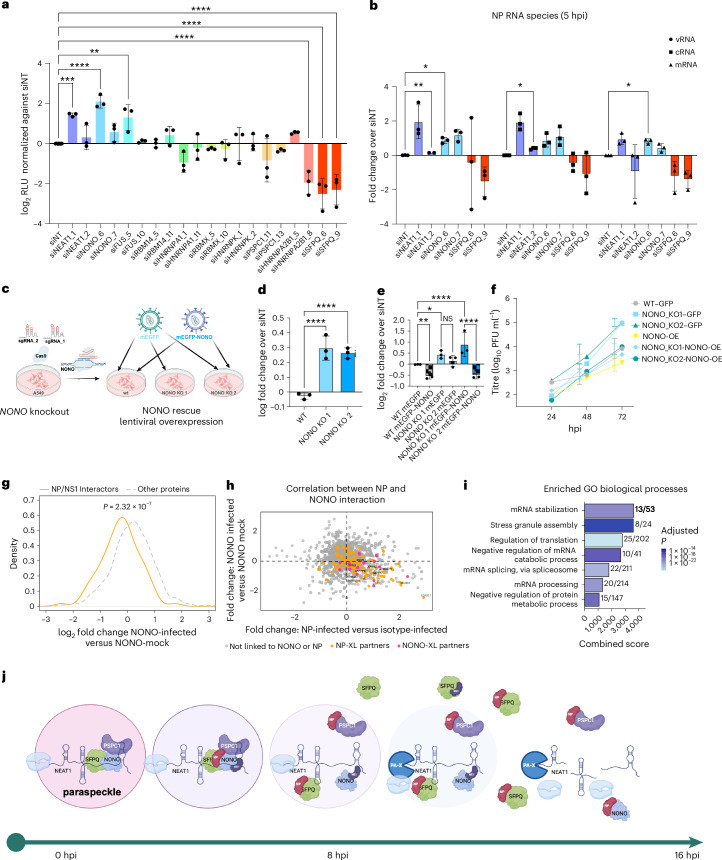
Functional analysis of paraspeckles during IAV infection. **a**, Luciferase activity in A549 cells transfected with paraspeckle-targeting siRNAs. Data are mean ± s.d. (*n* = 3). siNEAT1_1 and siNEAT1_2 indicate siRNAs targeting *NEAT1*. Some gene data were reproduced from Extended Data Fig. 6c. **b**, qPCR of vRNA species (NP fragment), normalized to *GAPDH*, in A549 cells with paraspeckle gene knockdowns after WSN infection (MOI 3, 6 hpi). Data are log_10_(fold change) relative to siNT, mean ± s.d. from *n* = 3 biological replicates; duplicate technical replicates were averaged. Statistics used a two-sided restricted maximum likelihood mixed-effects model with Geisser–Greenhouse correction. **c**, *NONO* KO and rescue workflow. **d**, Luciferase activity in wild-type (WT), NONO KO 1 and NONO KO 2 A549 cells infected with PB2-T2A-NanoLuc WSN (0.01 MOI, 48 hpi). Data are mean ± s.d. from *n* = 3 biological replicates; technical replicates were averaged. Statistics by ordinary one-way ANOVA with two-sided Dunnett’s test versus WT. **e**, Luciferase activity in WT, NONO KO 1, and KO 2 A549 cells overexpressing mEGFP or mEGFP–NONO (MOI 0.01, 48 hpi). mEGFP–NONO; mean ± s.d., *n* = 3. **f**, IAV growth kinetics in WT, NONO KO 1 and KO 2 A549 cells overexpressing mEGFP or mEGFP–NONO, infected with WSN (MOI 0.001). Data are mean ± s.d. from three independent infections. **g**, Density plot of log_2_ fold changes of LFQ values of proteins cross-linked to NONO, NP and NS1 identified by AP–MS (NONO infected versus NONO mock). NP/NS1-cross-linked proteins (orange) are codepleted versus non-cross-linked proteins (grey). *P* value by two-sided Wilcoxon rank-sum test. **h**, log_2_ LFQ fold changes of AP–MS interactors. *x* axis: NP AP–MS versus isotype-control AP–MS, both in infected cells. *y* axis: NONO AP–MS in infected versus mock cells; proteins cross-linked to NONO, NP or neither are shown in pink, orange or grey, respectively. **i**, Top 7 enriched Gene Ontology (GO) biological processes among SHVIP interactors, ranked by adjusted *P* value. Bar length, Enrichr combined score; bar colour, Benjamini–Hochberg-adjusted *P* value (darker = more significant). Numbers next to each bar indicate the number of SHVIP interactors annotated to the term/total genes in the term. Enrichment used Enrichr with a one-sided Fisher’s exact test and Benjamini–Hochberg correction. **j**, Schematic representation of paraspeckle disruption. In **a**, **b**, **e** and **f**, data are mean ± s.d.; two-sided ordinary one-way (**a**) or two-way (**e**,**f**) ANOVA with Dunnett’s multiple comparisons test. *P < 0.05, **P < 0.01, ***P < 0.001, ****P < 0.0001. Diagrams created in BioRender: **c**, Kotova, I. https://biorender.com/t41h404 (2026); **j**, Kotova, I. https://biorender.com/m6bdai8 (2026). cRNA, complementary RNA; NS, not significant; OE, overexpression. Source data

To evaluate the functional relevance of paraspeckle disruption during infection, we downregulated paraspeckle proteins by RNA interference and measured IAV replication by luciferase assay (Fig. 6a). Although knockdown of *HNRNPK* and *HNRNPA2B1* gave variable luciferase results, viral titration showed that *HNRNPK* depletion significantly reduced IAV replication (Supplementary Fig. 5a), consistent with previous reports^3^, whereas *HNRNPA2B1* knockdown increased replication, suggesting an antiviral effect. Consistent with our initial siRNA screen (Fig. 2h), *SFPQ* knockdown decreased IAV replication in A549 cells, confirming its role in enhancing IAV RNA processing^57,58^, whereas *NONO* knockdown had the opposite effect. *FUS* and *NEAT1* knockdown also had proviral effects (Fig. 6a). *NEAT1* and *NONO* knockdown substantially upregulated NP and HA vRNA, complementary RNA (cRNA) and mRNA by strand-specific quantitative PCR (qPCR)^65^ (Fig. 6b and Supplementary Fig. 5b–d), whereas *SFPQ* knockdown downregulated these vRNAs, aligning with its role in IAV RNA processing^57,58^. Only one siRNA targeting *NEAT1* increased replication in viral titration assays, further supporting its antiviral role (Supplementary Fig. 5e). *NEAT1* and *NONO* knockout (KO) impaired paraspeckle formation and increased IAV replication 2–2.5-fold (Fig. 6c,d and Supplementary Fig. 5f–m). Overexpressing mEGFP–NONO in wild-type cells impaired IAV replication by 30%, supporting an antiviral role for NONO (Fig. 6e). In *NONO* KO cells, mEGFP–NONO reduced viral replication, significantly in one clone but not the second (Fig. 6e), and reduced viral titres in both clones (Fig. 6f). No significant difference was seen for pdm09 (Supplementary Fig. 5n), but SC35M infection increased viral titres at 48 hpi in early-passage NONO KO cells (Supplementary Fig. 5o). A minimal *NEAT1_2* (ref. ^66^) construct partially rescued paraspeckle formation; low *NEAT1_2* expression (5%) probably prevented more complete rescue (Supplementary Fig. 5h).

Paraspeckle disruption was also proviral after *FUS* KO. *FUS* KO cells showed a variable increase in viral replication in luciferase assays (Supplementary Fig. 5p–q). Viral titration confirmed increased WSN replication across KO clones at 48 hpi (Supplementary Fig. 5r), whereas pdm09 was variable (Supplementary Fig. 5s).

To test whether paraspeckle-sequestered proteins become available for IAV interaction upon infection, we compared AP–MS targeting NONO in infected cells (disrupted paraspeckles) and non-infected cells (intact paraspeckles). Proteins cross-linked with NP or NS1 were depleted from the NONO interactome in infected cells (Fig. 6g and Supplementary Fig. 5t–v), without changes in overall protein levels in input samples (Supplementary Fig. 5w), suggesting redistribution from NONO to viral proteins. Consistently, the RNA-binding proteins hnRNPC, hnRNPA1, MATR3, SFPQ and hnRNPK were enriched in AP–MS targeting NP but depleted in AP–MS targeting NONO in infected cells (Fig. 6h,i). As regulators of paraspeckle integrity, RNA processing and transcriptional control^53^, their release from NONO and engagement with NP/NS1 may enhance viral replication, consistent with the proviral effects of SFPQ and reported roles of SFPQ and hnRNPK in IAV RNA splicing and replication^3,57,58,67^. SFPQ overexpression increased pdm09 titres (Supplementary Fig. 5y) and showed a similar, non-significant trend for WSN at 48 hpi (Supplementary Fig. 5z).

Together, these findings support a model in which IAV-induced paraspeckle disassembly releases proviral host factors recruited by viral proteins, such as NP and NS1, to promote replication (Fig. 6j).

## Discussion

Our in-cell XL-MS approach maps spatially resolved IAV–host PPIs in the native cellular environment. Unlike AP–MS and yeast two-hybrid studies using lysed cells, XL-MS preserves physiological interactions and late-stage-specific contacts. The dataset accordingly lacks earlier-stage PPIs, including RNA Pol II–RdRp^68^ and ANP32–PB2 (refs. ^22,69^). Instead, ANP32A/B leucine-rich repeat domains cross-linked with the M1 carboxy-terminal region, suggesting late-stage M1 may obstruct the ANP32A/B–RdRp interface and free vRNPs for nuclear export.

Our data traced HA maturation and export through the secretory pathway (Fig. 3b), recapitulating early ER folding, including the CALR/CANX cycle^70^ and PDIA3- and TMX1-mediated disulfide bond formation^41^. Combining in-cell XL-MS, loss-of-function perturbations, immunofluorescence and DQGlyco glycoprofiling, we identify three additional HA maturation host factors: the lectin cargo receptor LMAN2, the galactosyltransferase B4GALT1 and TM9SF2. DQGlyco analysis indicates that LMAN2 and B4GALT1 act site specifically on HA glycosylation, whereas TM9SF2 produced a site-unspecific phenotype with reduced total HA and a shift towards more extensively glycosylated forms. Both *TM9SF2* knockdown (Extended Data Fig. 7b) and overexpression^47^ impair virus production, suggesting that TM9SF2 levels must be tightly balanced. The role of TM9SF2 in HA maturation is consistent with reported functions of TM9SF2 in secretory trafficking dynamics, Shiga toxin trafficking^46^ and endosomal and lipid-associated sorting pathways^45^. These findings show that in-cell XL-MS resolves membrane-associated processes at suborganelle resolution and enables reconstruction of sequential steps of infection-related molecular pathways.

Guided by SHVIP, we found that IAV disrupts paraspeckles, membraneless organelles involved in stress responses and immune regulation^71,72^. These dynamic structures change in size, number and composition under stress^71,72^. Previous studies reported paraspeckle induction during hantavirus^73^, HSV-1 (ref. ^57^) and Dengue virus^74^ infection. In contrast, we consistently observed paraspeckle disassembly during IAV infection across tested strains and cell types (Fig. 4f–h). Although one study^57^ reported paraspeckle induction after IAV infection in HeLa cells, we also observed disruption in HeLa cells (Supplementary Fig. 4w–x). As HeLa cells are a suboptimal IAV model versus lung-derived cells^75^, this discrepancy may reflect differences in HeLa sublines or conditions, as we observed disassembly across multiple cell models, including primary human lung epithelial cells (HBEpCs).

We propose paraspeckle disassembly benefits IAV in two ways (Fig. 6j). First, it liberates host RNA-binding proteins needed for replication, including SFPQ, implicated in IAV RNA expression and possibly RNA synthesis and nuclear transport^57,58^, plus hnRNPK^3^, hnRNPC^61^ and hnRNPA1 (ref. ^60^). Consistently, depletion of some core paraspeckle components (NONO, FUS or NEAT1) increased infectious virus production (Fig. 6a and Supplementary Fig. 5a,e), whereas NONO rescue reversed this phenotype (Fig. 6e,f). SFPQ overexpression also enhanced viral replication (Supplementary Fig. 5y).

Second, paraspeckle disassembly may suppress antiviral responses. Given roles in stress adaptation and immune signalling, and our finding that NEAT1, NONO, FUS and hnRNPA2B1 restrict viral replication and transcription, loss of paraspeckles could attenuate host antiviral gene regulation^76^. We therefore posit that paraspeckle dismantling is an active viral strategy, not a secondary consequence of host transcriptional repression. Supporting this, paraspeckle-associated proteins were frequent NP and NS1 partners in our SHVIP dataset (Fig. 4a) and the most enriched host protein category targeted by IAV proteins (Extended Data Fig. 8). NP and NS1 specifically drove disassembly: either protein alone induced it (Fig. 5a–c), whereas other viral proteins had no detectable effect (Supplementary Fig. 4y–z). NEAT1 is possibly directly degraded by PA-X (Fig. 5d–f), while RNA Pol II inhibition (Fig. 5i–k) probably contributes later.

Although paraspeckle disruption was conserved across tested strains and cellular systems, the contribution of individual paraspeckle-associated proteins to viral replication differed between strains. NONO KO increased WSN replication but had little effect on pdm09 or the avian SC35M strain. FUS depletion increased replication of both WSN and pdm09, whereas SFPQ overexpression selectively affected pdm09 but not WSN. Thus, although paraspeckle disruption broadly increases host RNA-binding protein availability, individual IAV strains differentially exploit specific components of this released host factor pool. The SHVIP dataset captures these interactions at individual viral–host contact sites, providing a framework for future comparative analyses of strain-specific interaction patterns.

Although validating all PPIs and contact sites in our map is beyond a single study, this snapshot provides a foundation for follow-ups. Five viral proteins, including all three polymerase subunits, cross-linked with calcium homeostasis-related proteins. Individual knockdowns did not significantly affect infection (Extended Data Fig. 6), probably because of functional redundancy. These interactions align with reports linking calcium levels to influenza virus infection^77^, supporting calcium-mediated effects through viral interactions with calcium-binding proteins.

In-cell XL-MS of IAV–host interactions could be extended to other IAV strains and earlier infection stages to capture interaction dynamics, including those involving IAV RdRp during transcription^68,78^. Future structural prediction integrating cross-links as restraints could use XL-MS data to map the IAV–human protein interface.

In conclusion, our work provides a unique resource of native IAV–host PPIs and a blueprint showing how integrating in-cell XL-MS, functional analyses and structural modelling resolves the native spatial and structural organization of viral–host interfaces and uncovers mechanisms of viral subversion and host cell perturbation.

## Methods

### Immortalized cell lines

Human A549 (86012804; ECACC) and HEK293T (CRL-3216; ATCC) cells were cultured in DMEM (11960044; Gibco) supplemented with 10% FBS (0270106; Gibco), GlutaMAX (35050038; Gibco), sodium pyruvate (11360039; Gibco) and penicillin–streptomycin (15070063; Gibco) at 37 °C and 5% CO_2_. Calu-3 cells (no catalogue number available—provided by Petr Chlanda, Heidelberg University; originally from Prof. Ralf Bartenschlager, Heidelberg University) were maintained in the same medium with 20% FBS. MDCK (84121903; ECACC), MDCK.II (00062107; ECACC), MDCK-SIAT1 (05071502-1VL; Sigma-Aldrich) and MDBK (no catalogue number available—provided by Ervin Fodor, University of Oxford; Sir William Dunn School of Pathology Cell Bank stock) cells were grown in MEM supplemented with 10% FBS, GlutaMAX, sodium pyruvate and penicillin–streptomycin. MEFs (no catalogue number available—provided by Prof. Dr. Michael Bader, Max Delbrück Center) were cultured in DMEM with 10% FBS, GlutaMAX, sodium pyruvate, penicillin–streptomycin and non-essential amino acids (11140050; Gibco). HeLa (Kyoto; no catalogue number is available for this line, provided by the Mahamid laboratory, which had received them from the Sara Cuylen-Haering laboratory, originally from Daniel Gerlich, Institute of Molecular Biotechnology of the Austrian Academy of Sciences; the Gerlich laboratory reported this HeLa Kyoto stock as originally obtained from S. Narumiya, Kyoto University, RRID:CVCL_1922, authenticated by multiplex human cell line authentication testing) were cultured in DMEM (11960044; Gibco) supplemented with 10% FBS (0270106; Gibco), GlutaMAX (35050038; Gibco), sodium pyruvate (11360039; Gibco) and penicillin–streptomycin (15070063; Gibco) at 37 °C and 5% CO_2_. A549 and HEK293T cells were authenticated by short tandem repeat profiling. All cell lines were routinely tested for mycoplasma contamination and confirmed negative.

### Primary cells

Primary HBEpCs (C-12640, donor lot number 499Z012.1; Promocell) were maintained in Airway Epithelial Cell Growth Medium (C-21060; Promocell) and cultured according to the manufacturer’s instructions in Airway Epithelial Cell Growth Medium at 37 °C with 5% CO_2_. HBEpCs were maintained as undifferentiated submerged cultures and were not differentiated at an air–liquid interface. Primary cells were used at passages 2–6 for all experiments. All cell lines were routinely tested for mycoplasma contamination (MycoplasmaCheck; Eurofins).

### Virus production

WSN was produced in confluent MDBK cells infected at a multiplicity of infection (MOI) of 0.001 in DMEM containing 0.5% FBS. After 36 hours incubation at 37 °C and 5% CO_2_, when full cytopathic effect was observed, supernatants were collected, clarified by centrifugation (1,500*g*, 10 minutes, 4 °C) and aliquoted for storage at −80 °C. pdm09 and H3N2 were produced in MDCK cells infected at an MOI of 0.001 in serum-free DMEM supplemented with 0.2% BSA and 1 µg ml^−1^ TPCK-treated trypsin. After 36–48 hours incubation at 37 °C and 5% CO_2_ and confirmation of full cytopathic effect and successful HA assay, viral supernatants were clarified and stored as mentioned earlier.

Recombinant SC35M (H7N7) was generated by transfecting HEK293T cells with 8 plasmids (pHW2000) encoding all 8 influenza gene segments (1 μg each) and Lipofectamine 2000 (Invitrogen) according to the manufacturers’ protocol. After an incubation period of 6 hours, cell culture medium was removed and replaced with fresh DMEM. At 48 hours post-transfection, supernatants were collected to inoculate fresh MDCK. For a secondary infection, supernatants from previously infected MDCK cells were collected at 48 hours post-infection, stored at −70 °C and used for inoculation of fresh MDCK cells. After clear cytopathic effect development, supernatants were collected and processed as described earlier.

### Virus infection

Cells were infected with IAV strains A/WSN/33 (H1N1), A/California/07/2009 (pdm09, H1N1), A/Aichi/2/1968 (H3N2) or SC35M (mouse-adapted variant of the highly pathogenic avian influenza virus strain SC35 derived from A/Seal/Massachusetts/1/80 (H7N7)) at the indicated MOI. Before infection, virus inocula were diluted in infection medium consisting of serum-free DMEM supplemented with 0.2% BSA and antibiotics. Cells were washed once with PBS and incubated with the virus inoculum for 30 minutes at 4 °C, followed by an additional 30 minutes at 37 °C with gentle rocking to allow virus attachment and entry. For SC35M, cells were incubated with virus inoculum for 30 minutes at 37 °C with gentle shaking to allow even distribution of virus particles on the cell layer. After infection, the inoculum was removed and replaced with fresh infection medium.

For infections with A/WSN/33 and SC35M, infection medium did not contain trypsin. For infections with A/California/07/2009 and A/Aichi/2/1968, infection medium was supplemented with TPCK-treated trypsin (1 µg ml^−1^) as required for these strains to support multicycle replication. Infected cells were incubated at 37 °C and 5% CO_2_ and collected at the indicated time points for downstream analyses.

### Reconstitution of recombinant IAV overexpressing NanoLuc on PB2

A recombinant IAV expressing NanoLuc fused to PB2 was generated using a 12-plasmid reverse genetics system as described previously^34^. The system included eight plasmids encoding the vRNA segments and four expression plasmids for PB2, PB1, PA and NP under control of a human Pol I promoter. The NanoLuc reporter gene was inserted in-frame at the amino terminus of PB2, separated by a self-cleaving T2A peptide to allow independent translation.

### HA assay

The HA assay was performed in 96-well round-bottom plates using 2-fold serial dilutions of virus in PBS (50 µl per well). An equal volume (50 µl) of 0.5% chicken erythrocyte suspension was added to each well. Plates were incubated for 30 minutes at 4 °C, followed by 30 minutes at room temperature. HA titres were determined visually as the highest virus dilution producing complete haemagglutination, defined by a uniform lattice across the well. Results are expressed in haemagglutination units.

### Plaque assay

For WSN, confluent MDCK cells in 6-well plates were infected with 330 µl of serially diluted virus in infection media (DMEM with 0.2% BSA). Plates were incubated for 30 minutes at 4 °C, followed by 30 minutes at 37 °C with gentle rocking. Cells were overlaid with Avicel-containing media (DMEM, 0.2% BSA) and incubated for 72 hours. After removing the overlay, cells were washed with PBS and stained with 1% crystal violet for 5 minutes. Plaques were counted to calculate viral titre in plaque-forming units per millilitre (PFU ml^−1^). For pdm09 and H3N2, MDCK-II cells were infected as above and overlaid with DMEM containing Avicel, 0.2% BSA and 1 µg ml^−1^ TPCK-treated trypsin. After 72 hours, cells were fixed with 4% paraformaldehyde for 30 minutes at 4 °C and blocked with 0.3% hydrogen peroxide. Viral plaques were detected by immunostaining with anti-NP antibodies (Aichi, MA5-42364, Thermo Fisher; pdm09, ab43821, Abcam; 1:1,000) for 1 hour at room temperature, followed by HRP-conjugated secondary antibody and KPL TrueBlue substrate (15 minutes). Viral titres were calculated as PFU ml^−1^:

$$\mathrm{Titre}\,\left(\frac{\mathrm{PFU}}{\mathrm{ml}}\right)=\frac{\left(\mathrm{number}\,\mathrm{of}\,\mathrm{plaques}\,\times \,\mathrm{dilution}\,\mathrm{factor}\right)}{\mathrm{volume}\,\mathrm{of}\,\mathrm{virus}\,\mathrm{innoculum}\,(\mathrm{ml})}$$

### Luciferase assay

A549 cells were seeded at 1 × 10^5^ cells per well in 24-well plates and transfected 12 hours later in duplicate with 10 µM siRNAs using RNAiMAX (Thermo Fisher Scientific), according to the manufacturer’s instructions. After 72 hours incubation at 37 °C and 5% CO_2_, cells were washed with infection media and infected with PB2-T2A-NanoLuc recombinant WSN at an MOI of 0.01. At 48 hours post-infection, cells were lysed in 200 µl Passive Lysis Buffer (Promega) per well and incubated at room temperature for 20–30 minutes on a rocking platform. For luciferase activity, 25 µl of lysate was mixed with 25 µl NanoGlo substrate (Promega) in a white 96-well plate. Luminescence was measured after 5–10 minutes equilibration using a luminometer (1,000 ms integration time).

### Viral growth kinetics

For viral growth kinetics, cells were infected at low multiplicity to allow multicycle replication. A549 cells and HBEpCs were seeded at 2 × 10^5^ cells per well, transfected with the indicated siRNAs for 72 hours, and subsequently infected with WSN at an MOI of 0.001 or pdm09 at an MOI of 0.01. For experiments performed in KO or overexpressed cell lines, cells were seeded at 7.5 × 10^5^ cells per well before infection. Following infection, cells were washed and maintained in the appropriate infection medium.

At the indicated time points post-infection, 50 µl of supernatant was collected and stored at −70 °C until analysis. Viral titres were determined by plaque assay on MDCK cells (WSN) or MDCK-SIAT1 cells (pdm09) as described earlier. Growth curves are presented as PFU ml^−1^ at 24 hpi, 48 hpi and 72 hpi.

### FISH

Cells (1 × 10^5^ per well) were seeded onto sterilized glass coverslips in 24-well plates and grown to ~70–80% confluence. Cells were washed with DPBS, fixed in 4% paraformaldehyde for 10 minutes at room temperature and permeabilized in 70% ethanol overnight at −20 °C. Hybridization and amplification were performed using the hybridization chain reaction (HCR v.3.0) protocol adapted from ref. ^79^, with optimizations for detecting IAV vRNA, mRNA and antisense RNA (see Supplementary Table 5 for probe sequences and design). Briefly, fixed cells were prehybridized with Probe Hybridization Buffer (Molecular Instruments) and incubated overnight with 2 pmol of each probe at 37 °C. Following washes in Probe Wash Buffer and SSCT, amplification was carried out overnight at room temperature using snap-cooled HCR hairpins (h1 and h2). Cells were washed in 5× SSCT, stained with DAPI and mounted using Dako antifade mountant (Agilent). Samples were imaged using a confocal microscope with appropriate filter settings for DAPI and HCR fluorophores. Acquisition settings were optimized to reduce background and enhance signal detection.

### siRNA transfection

All siRNAs were purchased from Qiagen (Supplementary Table 6). A549 cells at ~60% confluence were transfected with 5 pmol siRNA using Lipofectamine RNAiMAX (13778075; Thermo Fisher Scientific) according to the manufacturer’s protocol. Knockdown efficiency was assessed 72 hours post-transfection by qPCR or western blot.

### PLA

PLA was performed using the Duolink In Situ PLA kit (Sigma-Aldrich) following the Sigma protocol. A549 cells were seeded on glass coverslips and infected as described earlier. At the indicated time point post-infection, cells were washed with PBS, fixed with 4% paraformaldehyde for 10 minutes at room temperature and permeabilized with PBS containing 0.2% Triton X-100 for 10 minutes at room temperature, according to the manufacturer’s instructions. Next, cells were blocked in the supplied blocking solution and incubated with the primary antibodies. After washing, PLA probes were applied, followed by ligation and rolling-circle amplification steps. PLA signals were detected using Duolink detection reagents coupled to Alexa Fluor 488. Nuclei were counterstained with DAPI, and coverslips were mounted using Dako mounting medium (Agilent). Samples were imaged by fluorescence microscopy using identical acquisition settings across conditions, and PLA puncta and signal intensity were quantified from maximum *z*-projection images.

The following primary antibodies were used for PLA: anti-IAV nucleoprotein (NP) (clone C43; Abcam), anti-NS1 (EPR28247-51; Abcam), anti-HA (clone 1.B.408; Abcam), anti-PB2 (PA5-32220; Thermo Fisher Scientific), anti-M1 (clone GA2B, MA1-80736; Thermo Fisher Scientific), anti-hnRNPK (F45P9C7; Thermo Fisher Scientific), anti-RAB11A (67902-1-Ig; Proteintech), anti-hnRNPA1 (67844-1-Ig; Proteintech), anti-NF45 (67685-1-Ig; Proteintech), anti-hnRNPA2B1 (67445-1-Ig; Proteintech), anti-CCAR2 (66497-1-Ig; Proteintech), anti-SFPQ (67129-1-Ig; Proteintech), anti-nucleolin (D4C7O, number 14574; Cell Signaling Technology), anti-B4GALT1 (PA5-52744; Thermo Fisher Scientific), anti-VIP36 (PA5-90437; Thermo Fisher Scientific), anti-hnRNPM (26897-1-Ig; Proteintech), anti-ERP29 (24344-1-AP; Proteintech), anti-GLUT1 (21829-1-AP; Proteintech), anti-RPL35 (14826-1-AP; Proteintech), anti-ERGIC-53 (13364-1-AP: Proteintech), anti-hnRNPC (11760-1-AP; Proteintech) and anti-ANP32B (10843-1-AP; Proteintech).

### Cell viability

Cell viability was measured using the CellTiter-Glo 2.0 Assay Kit (G9241; Promega). Treated cells in 24-well plates were incubated with reagent directly added to the culture medium, followed by 10 minutes of shaking at room temperature. Luminescence, proportional to intracellular ATP levels, was measured using a plate reader and normalized to untreated controls.

### CRISPR–Cas9-mediated KO cell lines

Single guide RNAs were designed using Benchling or purchased as validated guides (IDT) and resuspended at 1 µM. Recombinant Cas9 protein (EMBL Protein Production Facility) was diluted to 1 µM in Opti-MEM. A549 cells were seeded at 1.5 × 10^5^ cells per well in 6-well plates at <50% confluence to ensure high KO efficiency. Cas9–single guide RNA ribonucleoprotein (RNP) complexes were formed in Opti-MEM and transfected using Lipofectamine RNAiMAX. After 96 hours, cells were either collected for genomic DNA (NEB kit) or seeded at 0.5 cells per well in 96-well plates for clonal selection. Target loci were PCR-amplified, gel-purified and sequenced (Sanger). Indel frequency was analysed using TIDE, and KO was confirmed by western blotting.

### Plasmid transfection

Plasmid transfections were performed in HEK293T cells at 70–80% confluence using Lipofectamine 3000 (Thermo Fisher Scientific) according to the manufacturer’s instructions. Plasmid DNA and Lipofectamine 3000 reagent were each diluted in Opti-MEM (Thermo Fisher Scientific), combined and incubated for 15 minutes at room temperature to allow DNA–lipid complex formation. The complexes were added dropwise to the cells and incubated for 4–6 hours at 37 °C in 5% CO_2_, after which the medium was replaced with fresh complete DMEM. Cells were incubated for an additional 16–48 hours before analysis. Transfection efficiency was assessed by cotransfection with a GFP-expressing plasmid or by fluorescence microscopy following immunofluorescence or FISH staining.

### Lentiviral cell line generation

Lentiviral vectors encoding mEGFP-tagged NONO, PSPC1 or SFPQ were cotransfected into HEK293T cells with packaging plasmids using a calcium phosphate transfection protocol. Supernatants containing lentiviral particles were collected after 48 hours, filtered and stored at −70 °C. A549 cells were transduced with viral supernatants in the presence of polybrene and incubated for 48–72 hours to allow stable genomic integration. Transduced cells were selected using 10 µg ml^−1^ Blasticidin and maintained in complete DMEM. Expression of tagged proteins was confirmed by fluorescence microscopy and western blotting. Protein functionality was verified by their colocalization with NEAT1 in paraspeckles.

### RNA extraction, reverse transcription and qPCR

Total RNA was extracted using the RNeasy Kit (Qiagen) according to the manufacturer’s instructions. Cells were lysed in buffer RLT supplemented with 5 mM TCEP, and RNA was purified on RNeasy columns with RW1 and RPE wash buffers. RNA was eluted in RNase-free water, quantified using a NanoDrop spectrophotometer and stored at −70 °C.

Reverse transcription was performed with up to 2 µg RNA per 10 µl reaction using the High-Capacity cDNA Reverse Transcription Kit (4368814; Thermo Fisher Scientific). qPCR was carried out using PowerUp SYBR Green Master Mix (A25742; Thermo Fisher Scientific) with gene-specific primers (Supplementary Table 7). Thermal cycling and melt curve analysis were performed according to the manufacturer’s protocol. Relative gene expression was quantified using the ΔΔCt method and normalized to *GAPDH*.

### Immunoblotting

Whole-cell lysates were resolved by SDS–PAGE using 4–12% precast TG PRiME gels (SERVA) and transferred to nitrocellulose membranes (Bio-Rad). Membranes were blocked in 2.5% BSA (in TBS with 0.01% Tween-20) for 1 hour at room temperature, followed by overnight incubation at 4 °C with primary antibodies diluted in blocking buffer. After 3 washes in TBS-Tween, membranes were incubated for 1 hour at room temperature with HRP-conjugated anti-mouse or anti-rabbit secondary antibodies (1:5,000), washed again and developed using enhanced chemiluminescence (A38555; Thermo Fisher Scientific). Antibodies used for immunoblotting are listed in Supplementary Table 8. Uncropped blots are available in the Source data.

### Affinity pulldown of Twin-Strep-tagged proteins

HEK293T cells were transfected with Twin-Strep-tagged constructs (NONO, M2 or empty vector control) using PEI at a 1:3 DNA:PEI ratio. At 24 hours post-transfection, cells were collected by scraping in PBS, washed and lysed in IP lysis buffer (Thermo Fisher) supplemented with HALT protease inhibitor cocktail. Lysates were clarified (17,000*g*, 20 minutes, 4 °C) and incubated with Strep-Tactin magnetic beads (IBA Lifesciences) pre-equilibrated in Buffer W. After 1 hour incubation at room temperature with rotation, beads were washed and bound proteins were eluted in Laemmli buffer at 95 °C for 2 minutes. Eluates were analysed by SDS–PAGE and western blot.

### IP

A549 cells were seeded in 10 cm dishes (4 × 10^6^ cells per dish) and infected with WSN (MOI 3) for 14 hours. Cells were collected and lysed in IP buffer (Thermo Fisher) supplemented with HALT protease inhibitor (Thermo Fisher). For certain IPs (for example, NP, NONO, SFPQ and PSPC1), 0.1% SDS was added to reduce non-specific binding. Lysates were cleared by centrifugation (max speed, 20 minutes, 4 °C). IP was performed with 10 µg of antibody and 500 µl of cleared lysate for 4 hours at 4 °C, followed by incubation with Protein A/G or G Dynabeads (Thermo Fisher) for 1 hour at room temperature. Isotype-matched control antibodies were used in all experiments. Beads were washed with TBS + 0.05% Tween-20 and lysis buffer and finally with TBS alone. For AP–MS, bound proteins were eluted in 8 M guanidine HCl at 95 °C and precipitated in cold ethanol overnight. Inputs were processed by methanol–chloroform precipitation. For western blotting, elution was performed with 0.2 M glycine (pH 2.0), followed by Tris neutralization and/or SDS loading buffer. Eluates were analysed by SDS–PAGE, western blot or mass spectrometry as described later. All AP–MS experiments were performed in triplicate.

### SHVIP sample preparation

Cells were infected at an MOI of 5 and, at 5 hpi, incubated in labelling medium containing 500 µM L-homopropargylglycine (L-HPG) in methionine-free DMEM with 0.2% BSA. At 14 hpi, cells were washed with PBS and collected by scraping. For cross-linking, cell pellets were resuspended in PBS and cross-linked with 5 mM DSSO for 1 hour at room temperature with constant agitation. The reaction was quenched with 30 mM Tris (pH 8.0) for 20 minutes. Cells were lysed in buffer containing 200 mM Tris (pH 8.0), 4% CHAPS, 1 M NaCl, 8 M urea and protease inhibitors. Lysates were frozen at −80 °C until enrichment. After thawing, lysates were treated with Benzonase and sonicated (10 × 30 second on/off cycles; Bioruptor Pico) and then clarified by centrifugation (10,000*g*, 10 minutes). A 50 µl aliquot was saved as input. Enrichment of HPG-labelled proteins was performed as described previously^80^: 800 µl of lysate was incubated with 200 µl picolyl-azide agarose beads (Click Chemistry Tools), and copper(I)-catalysed click chemistry was performed overnight at room temperature with rotation. Proteins were reduced with 10 mM DTT (70 °C, 15 minutes) and alkylated with 40 mM chloroacetamide (room temperature in the dark). Beads were washed sequentially in gravity columns with: 1% SDS, 250 mM NaCl and 5 mM EDTA in 100 mM Tris (pH 8.0); 8 M urea in 100 mM Tris (pH 8.0); 80% acetonitrile (ACN) in water; 5% ACN in 50 mM triethylammonium bicarbonate (TEAB); and 5% ACN with 2 M urea in 50 mM TEAB. Bound proteins were digested on-bead overnight at 37 °C with trypsin and LysC in the final wash buffer. Peptides were collected, acidified with 1% formic acid, desalted using C18 stage tip (or Sep-Pak C8 columns for cross-linked peptides), dried by vacuum centrifugation and stored at −20 °C until LC–MS or further processing. Input samples were processed by methanol–chloroform precipitation and digested in buffer containing 1% sodium deoxycholate, 5 mM TCEP and 40 mM 2-chloroacetamide in 50 mM TEAB. The resulting peptides were desalted as described earlier.

### Off-line fractionation of cross-linked peptide samples

To enrich cross-linked peptides, HPG-enriched samples were first subjected to strong cation exchange (SCX) chromatography using a polysulfethyl A column. Peptides were separated with a 95-minute linear gradient and collected in 45-second intervals. Fractions were desalted, dried and stored at −20 °C. After initial measurement, selected SCX fractions were pooled and further separated by size exclusion chromatography (SEC) using a Superdex 30 column. Fractions were collected every 2 minutes, dried and stored at −20 °C before LC–MS/MS.

### LC–MS/MS of bottom-up proteomics and AP–MS samples

Bottom-up samples and AP–MS eluates were analysed using Orbitrap Fusion Tribrid or Exploris 480 instruments coupled to nano-LC systems. Peptides were loaded onto a 50 cm in-house packed C18 column and separated by 120–180 minute gradients.

For bottom-up and AP–MS samples, MS1 scans were acquired at 120,000 resolution, dynamic exclusion was set to 40 seconds, precursors (charge +2 to +4) were isolated (1.6 *m*/*z* window) and fragmented by higher-energy collisional dissociation (HCD) at 30% normalized collision energy (NCE), with MS2 detection performed in the ion trap (Fusion) or Orbitrap (Exploris).

### LC–MS/MS of cross-linked samples

Cross-linked peptides were analysed on an Orbitrap Fusion Lumos equipped with a FAIMS Pro Duo interface. MS1 scans were performed at 120,000 resolution using FAIMS voltages (−50 V, −60 V and −75 V). Precursors (charge +4 to +8) were fragmented by stepped HCD (21%, 27% and 33% NCE) and MS2 scans acquired in the Orbitrap at 60,000 resolution.

### Bottom-up proteomics data analysis

Raw files were searched using MaxQuant v.1.6.2.6a^81^ against the human SwissProt database (release 2020) and the IAV/WSN/1933 (H1N1) proteome. Trypsin was used for in silico digestion (max 2 missed cleavages). Carbamidomethylation (C) was set as fixed modification and variable modifications, including oxidation (M), acetylation (N-term) and methionine replacement with HPG, where appropriate. iBAQ and label-free quantification (LFQ) quantification were enabled. Contaminants, reverse hits and proteins identified only by site were removed. For LFQ, missing values were imputed as described previously^16^. Fold changes were log_2_-transformed and *P* values were computed using 2-sided *t* tests. All AP–MS experiments were performed in triplicates.

Raw data were searched using Scout (v.1.5.1)^82^ with DSSO cross-linker settings and residue specificity for K, S, T and Y. Search parameters included: minimum peptide length, 6; max missed cleavages, 3; precursor mass tolerance, 10 ppm; fragment mass tolerance, 20 ppm; static mod, carbamidomethylation (C); and variable mod, oxidation (M).

Data were filtered at 2% or 5% FDR on cross-link spectrum match, residue-pair and PPI levels. For performance evaluation (Extended Data Fig. 1m–r), SCX fractions were searched per replicate. For the full dataset, SCX and SEC files were combined for global analysis.

### Glycosylation analysis

For each condition, 5 million A549 cells were seeded in a 15 cm dish. After 12 hours, cells were transfected with siRNAs targeting B4GALT1 or LMAN2 (2 independent siRNAs per target) using Lipofectamine RNAiMAX (Thermo Fisher Scientific) according to the manufacturer’s instructions. At 72 hours post-transfection, cells were infected with WSN at an MOI of 3 as described earlier. At 14 hpi, cells were collected by scraping, washed with PBS and frozen.

Glycoproteomics experiments were performed as described previously^48^ with minor adjustments: after lysis, digestion and glyco-enrichment, the samples were labelled with tandem mass tag (TMT) reagents and pooled. The samples were prefractionated into 24 fractions. After analysis of the glycopeptide samples on a Exploris 480, generated raw files were searched using MSFragger v.4.0 in Fragpipe v.20.0 against the Swissprot *Homo sapiens* database (UP000005640, 20,443 entries) combined with the influenza A database (UP000009255, 13 entries).

For the quantitative analysis of proteomics and glycoproteomics data, we first normalized the TMT reporter intensities for glycopeptides and proteins with complete quantification across all conditions (that is, TMT reporter ion intensity different from zero for all channels) using the normalizeVSN function (variance-stabilizing normalization; VSN) of the limma R package. We corrected glycopeptide intensities per protein by removing abundance-derived intensity using a linear regression for all glycopeptides with a matched total protein intensity. The corrected and normalized glycopeptide intensities and the normalized protein intensities were used for the differential expression analysis using the limma R package. For the design of the differential expression analysis, we considered the knockdown condition and the sample replicate. Contrasts were set for the comparison between the non-targeting control and the respective knockdown conditions.

Glycosylation at the N27/N28 sites of HA could not be assessed, as the corresponding amino-terminal region (residues 3–43) lacks lysine and arginine residues and therefore does not generate detectable peptides under the trypsin-based proteomics and glycoproteomics workflows used in this study.

### Confocal microscopy

All imaging was conducted using a Nikon Ti2 microscope equipped with a CSU-W1 spinning disk confocal unit and an Andor DU-888 X-11633 camera. The microscope was controlled by NIS-Elements software and used a 100×/1.49 SR Apo TIRF AC oil immersion objective (Nikon). For live-cell imaging, cells were seeded in Ibidi μ-Slide 4-well chambers (NC0685967; Ibidi) and maintained at 37 °C with 5% CO_2_. Imaging was performed using the 405 nm (DAPI channel), 488 nm (GFP channel) and 561 nm (Cy5 channel) lasers, with exposure times optimized for each experiment. The camera was maintained at −69.4 °C, with a binning of 1 × 1 and a readout speed of 30 MHz. *Z*-stacks were captured with an interval size of 130.6 nm and images were acquired at a resolution of 1,024 × 1,024 pixels.

### Image analysis

All images were processed as maximum intensity projections. Nuclei (DAPI) and paraspeckles (*NEAT1_2*) were segmented using CellProfiler v.4.2.6 with the Otsu thresholding method. The CV of the *NEAT1_2* signal within nuclei and the number of segmented paraspeckles were used as independent metrics. Both metrics have their respective advantages and disadvantages. Although CV relies on more reliable nuclei segmentation, it is an indirect measure of paraspeckle integrity and does not provide precise structural information. In contrast, paraspeckle quantification directly assesses paraspeckle formation but is more error prone, particularly at later time points when the NEAT1_2 signal diminishes.

Line profile analysis and preparation of representative images were performed using Fiji (ImageJ v.2.16.0/1.54p).

### Subcellular localization analysis of viral protein interactors

To compare subcellular localizations of host interactors across studies, we compiled viral–host protein interactions from SHVIP (this study), refs. ^13,15^ and the meta-analysis in ref. ^26^. For all datasets, only viral proteins that were identified in SHVIP (PB1, PB2, NP, NS1, M1, M2, HA and NA) were considered.

Host protein localizations were annotated based on data from the Human Protein Atlas. Only primary localization assignments were retained. A simplified set of localization categories was defined that grouped terms as follows: plasma membrane, cytosol, cytoskeleton, vesicular system, ER, mitochondrion, nucleoplasm, nucleoli, nucleus (other) and other, followed by manual curation. For ambiguous or multilocalized proteins, the most specific localization according to UniProt was assigned.

For SHVIP, we included only high-confidence host interactors filtered at 2% FDR at both the cross-linking PPI levels. For ref. ^13^, host interactors were taken directly from their published AP–MS dataset in HEK293 cells expressing individual FLAG-tagged viral proteins. For ref. ^15^, we used the high-confidence filtered dataset provided by the authors, based on AP–MS of 13 IAV proteins expressed in 3 cell types across 3 different IAV strains. For ref. ^26^, only host interactors identified in at least three independent studies or databases were considered, as described in their meta-analysis.

Viral protein localization was manually curated from published literature (Supplementary References) and database annotations (UniProt). Where conflicting evidence for localization existed, fractional assignments were split evenly across compartments (for example, a score of 1 distributed as 0.5 and 0.5 across 2 compartments).

### Validation of cross-links by host structures and AlphaFold 2 models

For each cross-linked human protein, all available structures in the PDB were retrieved using the corresponding UniProt identifier via the UniProt^83^ REST API and RCSB PDB Search API^84^. All cross-linked pairs were mapped to the retrieved structures, and if the corresponding pair was found among structures, Cα–Cα was calculated. If one pair was mapped to multiple structures, the shortest Cα–Cα distance was taken. The same mapping procedure was used for the random cross-linked pairs generated by taking arbitrary lysine–lysine pairs for individual proteins and protein pairs detected in PPIs. AlphaFold 2 (ref. ^44^) models were built using AlphaPulldown^85^. Structural figures were rendered using UCSF ChimeraX^86^.

### Virus–host interaction structural modelling

AlphaFold 2 models were built using AlphaPulldown^85^ v.2.0 using 24 recycles and 4 predictions per model. The sequence and template databases as of August 2024 were used. AlphaFold 3 was run using the original AlphaFold 3 code and using the default sequence database (downloaded in November 2024) and PDB database from November 2021 using the download script provided in AlphaFold 3 (ref. ^31^). AF3x^32^, a modelling protocol implemented within the AlphaFold 3 code that allows incorporation of cross-links as restraints in the form of explicit covalent ligands, was run with default settings and using version #dfb94a3 and the same databases as AlphaFold 3 (ref. ^31^). For AlphaFold 3 and AF3x, 20 random seeds were used per prediction (in some cases up to 1,000). The quality of models was assessed using the interface-predicted template modelling (ipTM) score, predicted template modelling (pTM), residue’s predicted local distance difference test (pLDDT) score and predicted alignment error, as returned by all the above tools. GRASP^33^ was run in normal mode using version 2a381bc, with four predictions per model, ranking by pTM. The multiple sequence alignment (MSA) and templates were the same as for AlphaFold2. Distance restraints in GRASP were applied using a 30 Å threshold and 0.05 FDR. Structural figures were rendered using UCSF ChimeraX^86^.

### Statistical analysis

Statistical analyses, plotting and graph preparation were performed using GraphPad Prism 10 (GraphPad Software). Exact *P* values for statistical analyses depicted in the figures are provided in the Source data.

### Other bioinformatics analyses

Functional enrichment analysis was performed using the Enrichr web platform^87^. The default Fisher’s exact test with the Benjamini–Hochberg method for correction for multiple hypotheses testing was used. Overlap with prior host–host interactions was calculated using BioGRID v.2.0.18 (ref. ^88^). For cross-links to viral proteins where alternative proteins with identical cross-linked peptide sequences were detected ambiguously (for example, histone proteins), only the first protein from the list of ambiguous candidates was selected for the analysis. Cross-link diagrams were drawn using xiNET^89^.

### Reporting summary

Further information on research design is available in the Nature Portfolio Reporting Summary linked to this article.

## Supplementary information

Supplementary Information Supplementary Note 1, Figs. 1–5, captions for Tables 1–8 and References.

Reporting Summary

Supplementary Data 1 Workbook combining Supplementary Tables 1–8 (cross-linking dataset, PPI overlap, functional enrichment, AlphaFold scores, FISH probe sequences, siRNAs, qPCR primers and antibodies) and source data for Supplementary Figs. 1–5.

Supplementary Video 1 NONO or SFPQ cellular localization in A549 cells stably overexpressing mEGFP-NONO or mEGFP-SFPQ, infected with WSN at MOI 3 or mock-infected, imaged every 40 minutes (maximum-intensity projection).

## Source data

Source Data Fig. 2 Statistical source data. Source Data Fig. 3 Statistical source data. Source Data Fig. 4 Statistical source data and per-panel statistics. Source Data Fig. 5 Statistical source data. Source Data Fig. 6 Statistical source data. Source Data Extended Data Fig. 1 Statistical source data. Source Data Extended Data Fig. 3 Statistical source data. Source Data Extended Data Fig. 5 Statistical source data. Source Data Extended Data Fig. 6 Statistical source data. Source Data Extended Data Fig. 7 Statistical source data. Source Data Extended Data Fig. 8 Statistical source data.

## Data Availability

Structural models have been deposited in Zenodo at https://zenodo.org/records/18053782 (ref. ^90^). Fluorescence images have been deposited in the BioImage Archive under accession code S-BIAD3316 (https://www.ebi.ac.uk/biostudies/bioimages/studies/S-BIAD3316). The MS cross-linking data have been deposited on the ProteomeXchange Consortium via the PRIDE^91^ partner repository with the dataset identifier PXD071226. The MS glycoproteomics data have been deposited in the ProteomeXchange Consortium via the PRIDE^91^ partner repository with the dataset identifier PXD072851. Source data are provided with this paper.
