# Supplementary material for: Mapping in-cell protein contact sites reveals hijacking of paraspeckles during influenza A virus infection
Source: Nat Microbiol. 2026 Jul 20;11(8):2247–65. doi: 10.1038/s41564-026-02416-1 (PMC13423814; doi:10.1038/s41564-026-02416-1)
Supplement: Supplementary file 1 — Supplementary Note 1, Figs. 1–5, captions for Tables 1–8 and References. [file 41564_2026_2416_MOESM1_ESM.pdf]

nature microbiology

Supplementary information https://doi.org/10.1038/s41564-026-02416-1

Mapping in-cell protein contact sites reveals hijacking of paraspeckles during influenza A virus infection

In the format provided by the authors and unedited

Supplementary Note 1 Expanded description of M2 interaction with LAT1

LAT1[1], a dimer of SLC7A5 and SLC3A2, is a membrane transporter complex primarily responsible for the uptake of large neutral amino acids into cells, playing a crucial role in cellular metabolism and growth. SLC7A5 forms the catalytic subunit, while SLC3A2 acts as the heavy chain, stabilising the complex at the plasma membrane. LAT1 is essential for amino acid uptake and mTORC1 activation, processes supporting cell growth and survival[1,2]. The heterodimeric nature of LAT1 enables efficient transport of substrates such as leucine, phenylalanine, and other branched-chain or aromatic amino acids.

In this study, SLC3A2 cross-linked to M2 with two cross-links detected at 2% FDR (Supplementary Fig. 2a) and its knockdown caused a small increase but was statistically significant only for one of the two siRNAs (Fig. 2h). A cross-link from M2 to SLC7A5 was also detected in the 5% FDR dataset (Supplementary Fig. 2a, Supplementary Table 1e–g). All cross-links are consistent with the orientation of LAT1 and M2 across the membrane (Supplementary Fig. 2a). Consistently, proximity ligation assays (PLA) revealed a strong PLA signal between M2 and both LAT1 subunits, SLC3A2 and SLC7A5 (Fig. 2a,b,f, Supplementary Fig. 1), supporting close spatial proximity of these proteins in cells. SLC3A2 knockdown led to a modest increase in viral infection in the reporter assay at 48 hpi (Supplementary Fig. 2b-c). By contrast, depletion of SLC7A5 significantly enhanced multi-cycle IAV replication at 48 and 72 hpi in A549 cells (Supplementary Fig. 2d). In the specific case of LAT1, interpretation of knockdown phenotypes is complicated by its heterodimeric architecture, comprising the transport-competent subunit SLC7A5 and the chaperone subunit SLC3A2, which is required for SLC7A5 trafficking to the plasma membrane[1]. SLC3A2 is typically more abundant and additionally serves as a chaperone for multiple other transmembrane proteins[1], such that its depletion may elicit broader cellular effects, while residual SLC3A2 levels may still stabilise functional LAT1 complexes. Consistent with this, western blot analysis revealed differential effects of SLC3A2 knockdown on SLC7A5 abundance (Supplementary Fig. 2b), providing a mechanistic explanation for the siRNA-dependent phenotypic variability and the absence of a robust increase in infectious virus production upon SLC3A2 depletion, in contrast to the consistent phenotype observed following SLC7A5 knockdown. These subunit-divergent effects may be explained by the broader functional repertoire and interaction network of

SLC3A2 in the cellular context or by residual SLC3A2 levels that were sufficient to stabilise the SLC7A5 heterodimer.

To validate the M2-LAT1 interaction, we performed an AP-MS experiment in infected cells comparing pull-down against SLC7A5 and M2 to isotype-matched specificity controls. This revealed the mutual enrichment of M2 and SLC7A5 (Supplementary Fig. 2e,f). Co-localisation of M2 and LAT1 at the plasma membrane was observed in A549 (Supplementary Fig. 2g-i) and primary human bronchial epithelial cells (HBEpCs) (Supplementary Fig. 2j-o), indicating that the interaction between M2 and LAT1 subunits occurs at cellular membranes and is preserved in the physiological context of primary cells. These findings suggest potential roles of this interaction in regulating amino acid transport or M2 localisation.

To assess whether the LAT1 complex contributes to early stages of IAV replication, we first analysed viral polymerase activity using a NanoLuc reporter virus at 5 hpi upon LAT1 genetic perturbation. Knockdown of SLC7A5 had no detectable effect on reporter activity, whereas depletion of SLC3A2 resulted in a modest but statistically significant reduction compared to siNT controls (Supplementary Fig. 2p).

To further examine whether depletion of LAT1 complex components affects viral protein accumulation at later stages of infection, we analysed NP expression by immunofluorescence at 4, 8, and 12 hpi (Supplementary Fig. 2r-s). While NP signal increased over time in all conditions, no pronounced qualitative differences in NP localisation or accumulation were observed upon knockdown of either SLC7A5 or SLC3A2 compared to controls (Supplementary Fig. 2r-s).

Together, these data indicate that the interaction between M2 and the LAT1 complex does not strongly impact early viral replication but may modulate viral fitness at later stages of infection, consistent with a subtle, context-dependent role of LAT1 during IAV replication.

REFERENCES

1. Napolitano, L., Scalise, M., Galluccio, M., Pochini, L., Albanese, L.M., and Indiveri, C. (2015). LAT1 is the transport competent unit of the LAT1/CD98 heterodimeric amino acid transporter. Int. J. Biochem. Cell Biol. *67*, 25–33. https://doi.org/10.1016/j.biocel.2015.08.004.

2. Xia, P., and Dubrovska, A. (2023). CD98 heavy chain as a prognostic biomarker and target for cancer treatment. Front. Oncol. *13*, 1251100. https://doi.org/10.3389/fonc.2023.1251100.

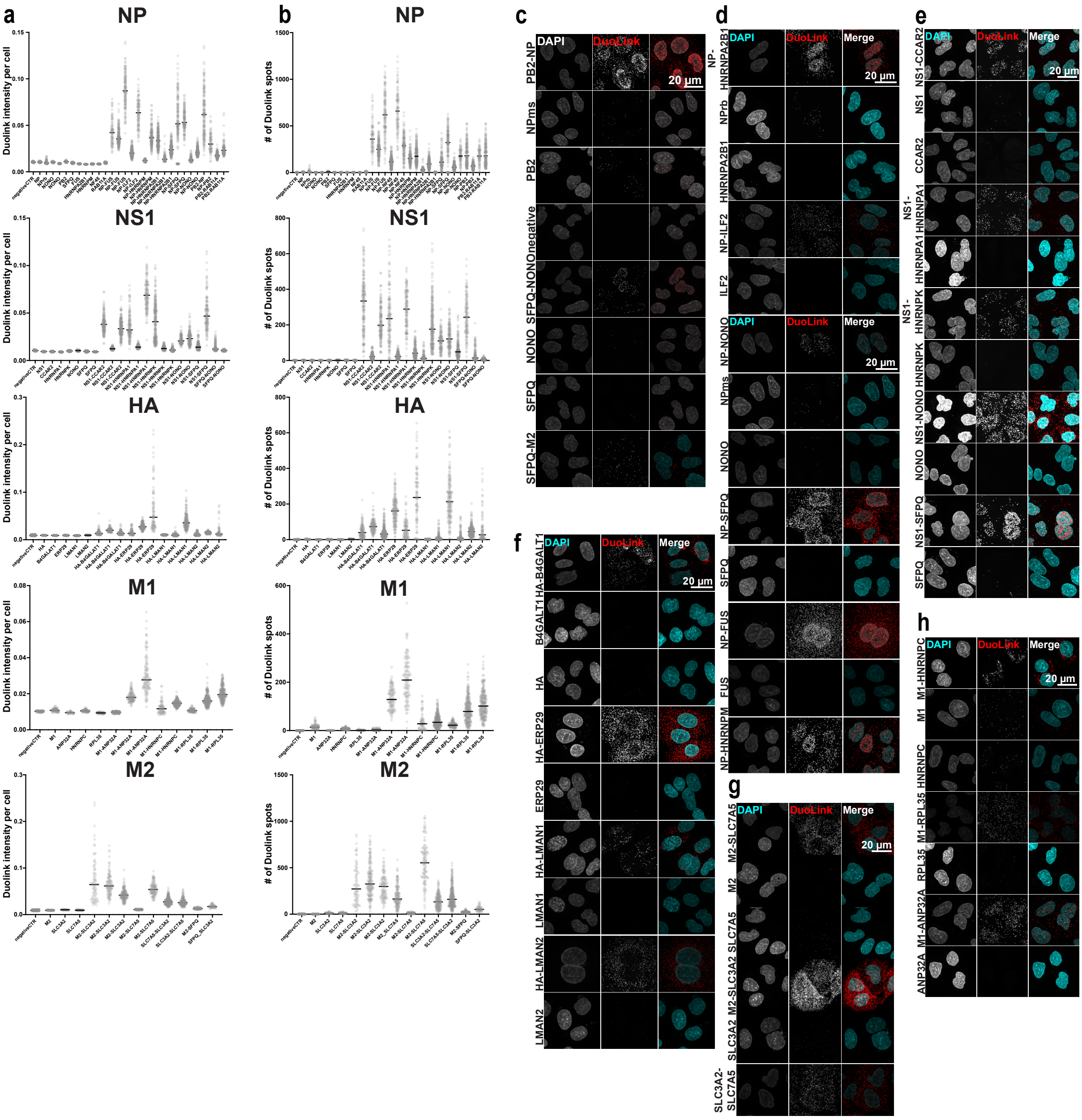

Supplementary Fig. 1 | *In situ* validation of selected viral–host interactions by proximity ligation assay (full data and including controls). a, Quantification of PLA signal intensity per cell for indicated viral proteins (NP, NS1, HA, M1, M2) paired with selected host factors, including single-antibody controls; individual replicates shown. Each dot represents one cell; data from at least two independent experiments shown with replicates displayed separately. **b,** Quantification of the number of PLA (Duolink) spots per cell for the same viral–host pairs and single-antibody controls shown in **(a).** Each dot represents one cell; data from one of at least two independent experiments shown with replicates displayed separately. **c,** Representative PLA images showing nuclear and cytoplasmic localisation of PLA signals for positive (PB2-NP and SFPQ-

NONO) and negative (SFPQ-M2) controls. DAPI (grey) and PLA signal (red). Representative of one of at least two independent experiments. Scale bar, 20 μm. **d-h,** Representative PLA images for selected viral–host protein pairs as in Fig. 2c-g with corresponding single-antibody controls. For each condition, DAPI (nuclei), PLA signal (red) and merged images shown. ms, mouse; rb, rabbit. Representative of one of at least two independent experiments. Scale bars, 20 μm.

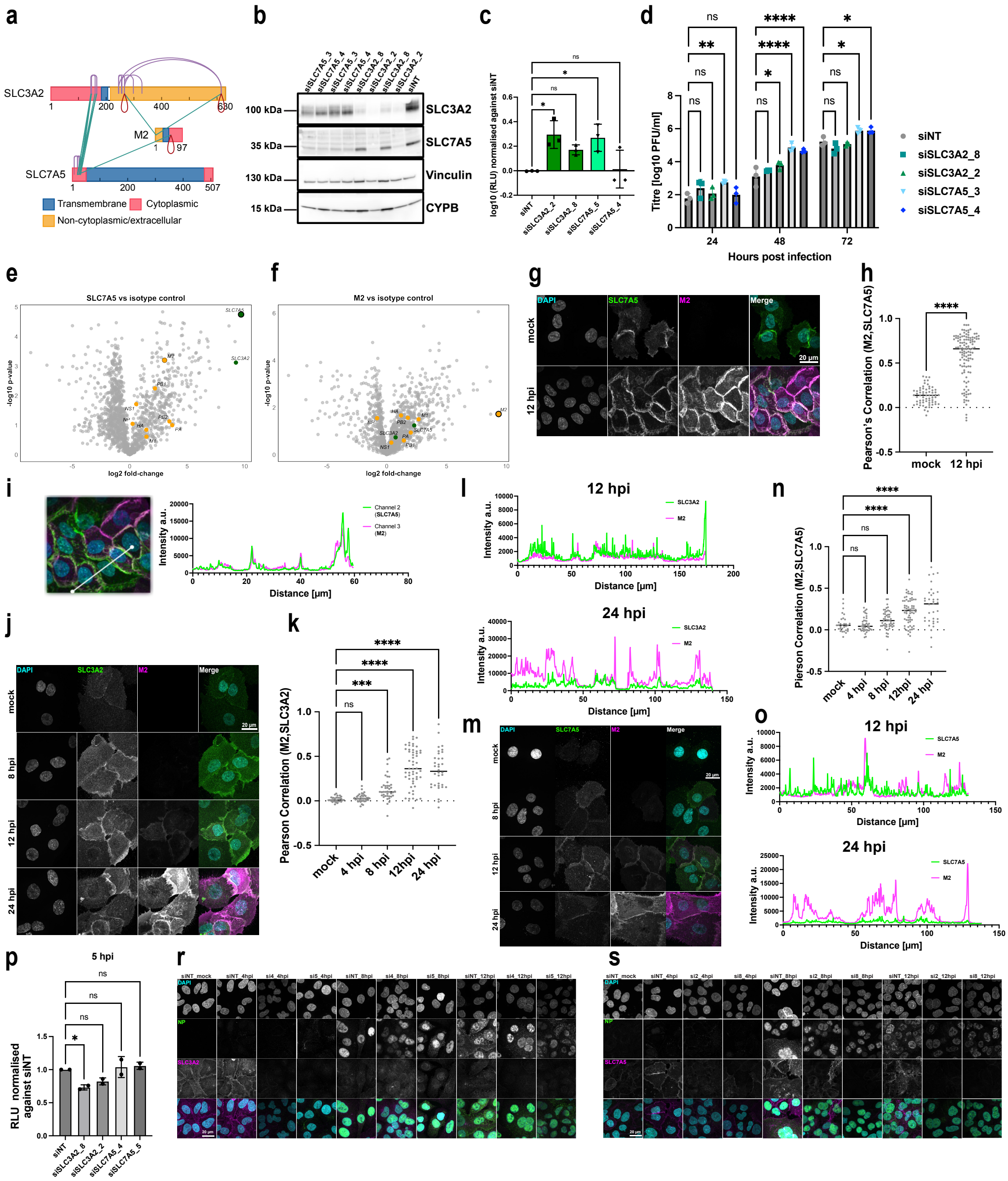

Supplementary Fig. 2 | LAT1 in IAV infection. a, Cross-linking network of M2 and LAT1, with two cross-links to SLC3A2 detected at 2% FDR and one cross-link between M2 and SLC7A5 detected at 5% FDR. **b,** Western blot analysis confirming knockdown of *SLC3A2* and *SLC7A5* in A549 cells. Vinculin and CYPB were used as loading controls. Two independent siRNAs were used per gene, and each condition was tested in at least two independent experiments with similar results. Representative blots are shown. **c,** Impact of *LAT1* knockdown on infection presented as Log(RLU) normalised to siNT at 48hpi. Data are represented as mean ± SD (n = 3), with individual experiment means shown as circles. Statistical analysis was performed using ordinary one-way ANOVA, two-sided. Statistical significance: *p<0.05, **p<0.01, ***p<0.001, ****p<0.0001. siSLC3A2 is replicated from Fig. 2h. **d,** Time course of WSN (MOI 0.001) viral growth kinetics in *SLC3A2* and *SLC7A5* knockdown cells. Supernatants were collected at the indicated time points and titres were determined

by plaque assay. Data represent mean ± SD from n = 3 independent biological replicates (independent infections). Each replicate corresponds to one independently infected culture (unit of study). **e, f,** Volcano plots showing log2 fold change versus statistical significance (-log10 P-value) for SLC7A5 (e) and M2 (f) versus isotype control. Viral proteins are depicted in orange, and SLC7A5 and SLC3A2 in green. P-values were calculated using two-sided Student's t-tests across n = 3 independent biological replicates (independent AP-MS experiments). P-values are unadjusted. **g,** Maximum intensity projection (MaxIP) of confocal images from A549 cells infected with WSN (Multiplicity of infection (MOI) 3) or mock at 12 hpi. Cells were stained for SLC7A5 (green), M2 (magenta), and nuclei (DAPI, cyan). Representative of at least two independent experiments with similar results. Scale bars, 20 μm. **h,** Pearson's correlation coefficient quantification of SLC7A5 and M2 colocalisation in membrane regions. Analysis was performed in mock-infected cells (n = 64) and WSN-infected cells at 12 hpi (n = 119). Statistical significance was determined using the Mann-Whitney test (****p<0.0001), two-sided. **i,** Representative plane and intensity profile showing the co-localisation of SLC7A5 and M2 in A549 cells at 12 hpi. **j,** Maximum intensity projection of primary HBEpCs showing colocalisation of SLC3A2 (green) and M2 (magenta). Images were taken at different timepoints during infection and show the distribution of SLC3A2 and M2 in the cells. The maximum intensity projection was generated from z-stacks to visualise overall protein localisation. Representative of at least two independent experiments with similar results. **k,** Pearson correlation coefficient between SLC3A2 and M2 in both the membrane and cytoplasm of HBEpCs. Correlation was calculated to assess the degree of colocalisation between these two proteins using Cell Profiler with 15% threshold. Statistical analysis was performed using a Kruskal–Wallis test (P < 0.0001) followed by Dunn's multiple comparisons. Significant increases were observed at 8, 12, and 24 hpi compared to mock (***P = 0.0002, ****P < 0.0001). No significant difference was observed at 4 hpi. n = 31–54 cells per condition. **l,** Intensity profile of a representative plane showing the localisation of SLC3A2 and M2 in several HBEpCs at 12 and 24 hpi. The profile represents the fluorescence intensity across a cross-section of the cells. **m,** Maximum intensity projection of HBEpCs showing colocalisation of SLC7A5 (green) and M2 (magenta). Images were taken at different time points during infection and show the distribution of SLC7A5 and M2 in the cells. The maximum intensity projection was generated from z-stacks to visualise overall protein localisation. Representative of at least two independent experiments with similar results. **n,** Pearson correlation coefficient between SLC7A5 and M2 in both the membrane and cytoplasm of HBEpCs. Correlation was calculated to assess the degree of colocalisation between these two proteins using Cell Profiler with 15% threshold. Statistical analysis was performed using a Kruskal–Wallis test (P < 0.0001) followed by Dunn's multiple comparisons. Significant increases in colocalisation were observed at 12 and 24 hpi compared to mock (****P < 0.0001), while changes at 4 and 8 hpi were not significant. n = 31–68 cells per condition. **o,** Intensity profile of a representative plane showing the localisation of SLC7A5 and M2 in several HBEpCs at 12 and 24 hpi. The profile represents the fluorescence intensity across a cross-section of the cells. **p,** Impact of LAT1 knockdown on replication of WSN (MOI1) in A549 cells at 5 hpi, presented as Log(RLU) normalised to siNT. Data are represented as mean ± SD (n = 3), with individual experiment means shown as circles. Statistical analysis was performed using ordinary one-way ANOVA, two-sided. **r, s,** Maximum intensity projection of fluorescent images of viral NP expression in A549 cells following siRNA-mediated knockdown of *SLC3A2* (r) or *SLC7A5* (s). Cells were infected with IAV and fixed at 4,

8 and 12 hpi. NP was detected by immunostaining; nuclei were counterstained with DAPI. Representative of at least two independent experiments with similar results. Exact P-values are provided in the Source Data.

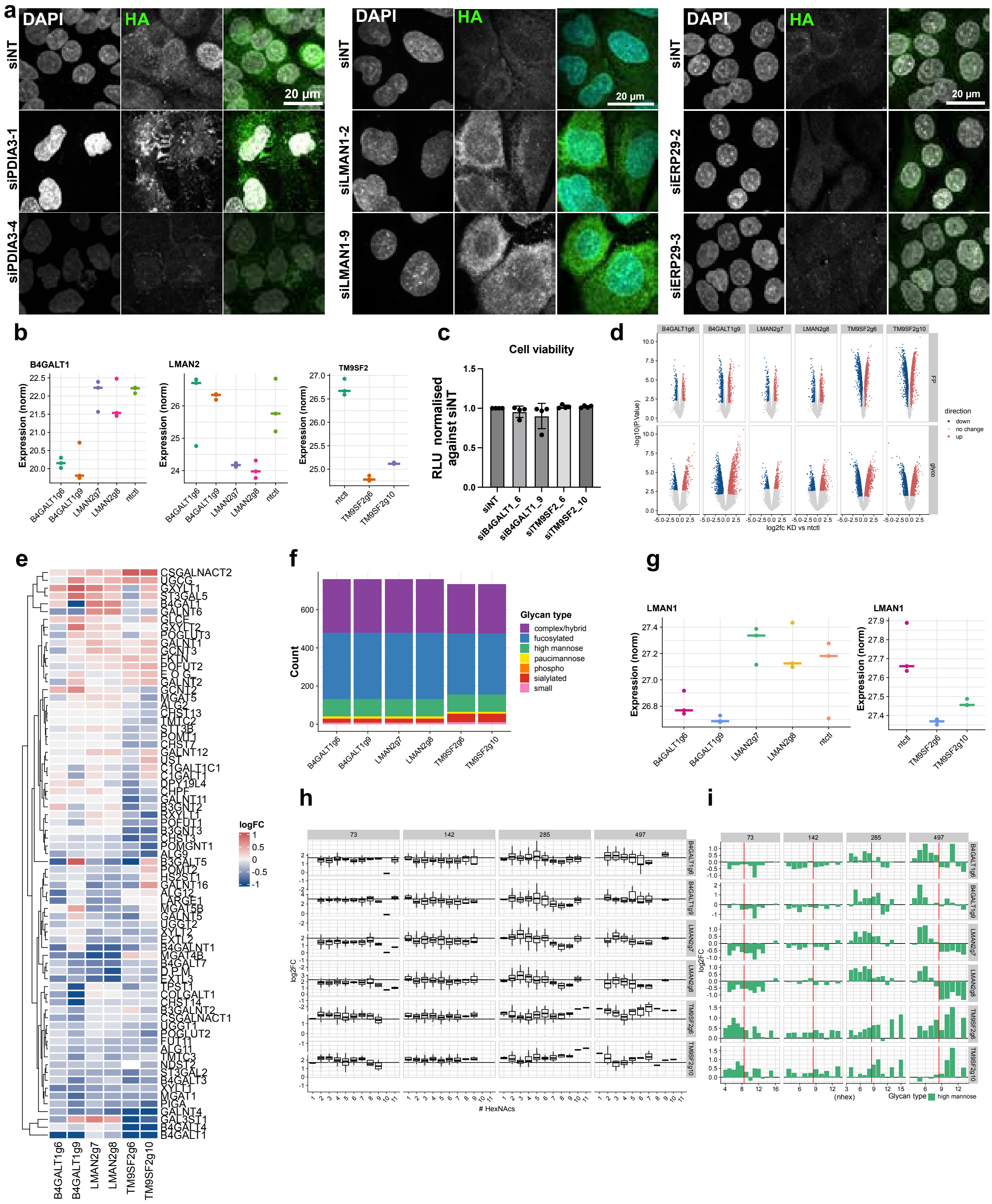

Supplementary Fig. 3 | Role of ER and Golgi proteins in IAV infection. a, HA localisation in A549 cells after siRNA knockdown of *PDIA3*, *LMAN1* and *ERP29* and WSN infection (MOI 3, 14 hpi). Two siRNAs per gene; experiments were repeated at least twice with similar results. Representative images shown. Scale bars, 20 µm. **b,** Protein abundance of selected ER and Golgi factors after knockdown in WSN-infected A549 cells (MOI 3, 14 h), shown as VSN-normalised log2 TMT intensities from the full-

proteome dataset; horizontal bars indicate the median of n=3 biological replicates. **c,** Cell viability 72 h after knockdown of indicated host factors, measured by CellTiter-Glo 2.0 and normalised to siNT. Data are mean ± SD from n=4 biological replicates, each averaging two technical replicates; significance was assessed by two-way ANOVA. **d,** Global proteome and glycoproteome changes after knockdown of ER and Golgi proteins. Volcano plots show log2 fold changes of TMT-based protein (full proteome, FP) or glycopeptide (glyco) intensities versus siNT and −log10 unadjusted P values (limma moderated t-test, empirical Bayes). Coloured points pass both Benjamini–Hochberg-adjusted $P < 0.05$ and $|\log_2 \text{fold change}| > \log_2(1.2)$; the horizontal cutoff near $-\log_{10}(P) \approx 2.5$ reflects the raw P corresponding to adjusted P ≈ 0.05. **e,** Heatmap of log2 fold changes in detected glycosylation enzymes across knockdowns. **f,** Distribution of glycan types across the full proteome after knockdown of ER and Golgi proteins, summarised into major classes. **g,** LMAN1 abundance across knockdown conditions, shown separately to assess compensatory or off-target effects after depletion of related ER–Golgi trafficking components. **h,** HA glycan maturation after knockdowns. Log2 fold changes of HA glycopeptides are plotted against increasing N-acetylhexosamine (HexNAc) residues for individual glycosylation sites. Box plots show median, interquartile range, whiskers to 1.5× the interquartile range, and outliers; points represent glycopeptides. Data are from n=3 independent infections, each analysed as a separate MS experiment. No statistical testing was performed. i, High-mannose glycan profiles at HA glycosylation sites after knockdown of *LMAN2*, *B4GALT1* and *TM9SF2*. Log2 fold changes versus siNT are plotted by hexose number (nHex). Red lines mark the approximate transition between less-trimmed ER-associated and more-trimmed Golgi-processed species.

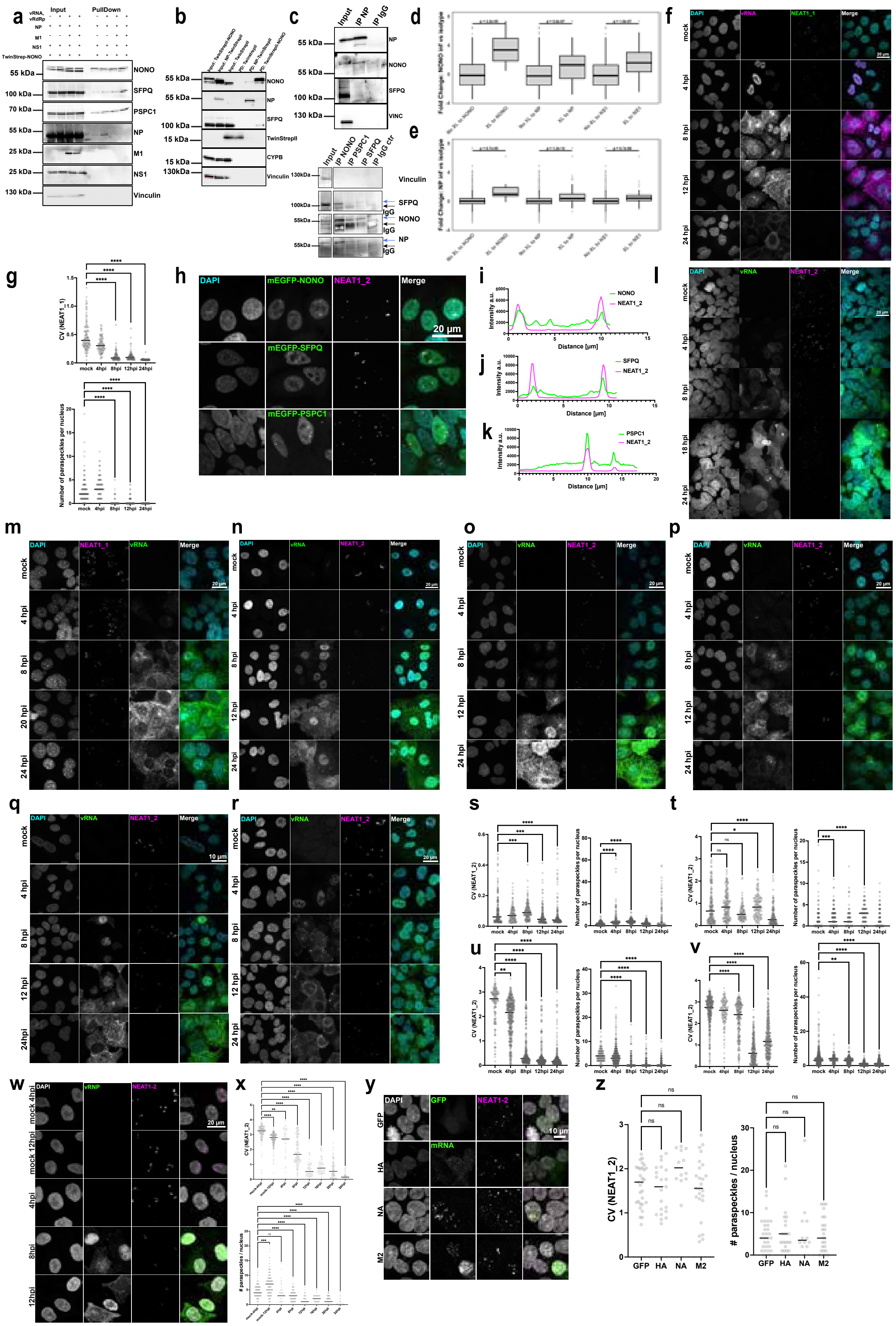

a
b
c
d
e
f
g
h
i
j
k
l
m
n
o
p
q
r
s
t
u
v
w
x
y
z
NONO
SFPQ
PSPC1
NP
M1
NS1
Vinculin
DAPI
vRNA
NEAT1_1
NEAT1_2
Merge
mock
4 hpi
8 hpi
12 hpi
24 hpi
CV (NEAT1_2)
Number of paraspeckles per nucleus
Distance [µm]
GFP
HA
NA
M2

Supplementary Fig. 4 | Analysis of paraspeckles during IAV infection. a, TwinStrep-tagged NONO was overexpressed in HEK 293T cells alongside the viral proteins NP, M1, and NS1, or the vRNP complex for 24 hours. Streptactin beads were used for pull-down to isolate the TwinStrep-NONO complex. Input and pull-down samples were analysed by western blot. Antibodies against NONO, SFPQ, and PSPC1 were used to confirm successful pulldown of paraspeckle proteins. Antibodies against viral proteins NP, M1, and NS1 were employed to assess their interaction with the NONO complex. **b,** StrepII pull-down of NONO and NP co-expressed in HEK293T cells using different tag orientations. HEK293T cells were transfected with: (1) HA-NONO + NP-TwinStrepII, (2) TwinStrepII-NONO + NP, or (3) NP + HA-NONO + TwinStrepII alone (negative control). StrepII pull-downs were performed, and both input and PD fractions were analysed by Western blot using the indicated antibodies. CYPB and Vinculin served as negative controls to monitor unspecific binding and loading. **c,** Immunoprecipitation of NP and IgG control in A549 infected cells (WSN, MOI=3, 14 hpi) (above). Immunoprecipitation of paraspeckle proteins NONO, SFPQ and PSPC1 and IgG control in A549 infected cells (WSN, MOI=3, 14 hpi) (below). The proteins of interest are annotated with the blue arrows and the IgG bands with the black ones. Vinculin was used as a negative control. **d**, AP-MS analysis of NONO versus isotype control in A549 cells (WSN, MOI 3, 14 hpi), showing log2 fold changes of protein LFQ values for enrichment of NONO, NP and NS1 cross-linked partners and those not cross-linked. Data are derived from n = 3 independent biological replicates (independent infections), each corresponding to one AP-MS experiment (unit of study). Box plots show the median (centre line), interquartile range (box; 25th–75th percentiles), and whiskers extending to 1.5× the interquartile range; points outside this range are shown as individual outliers. Individual points represent proteins. Statistical significance was assessed using a two-sided Wilcoxon rank-sum test. Significance levels: ***p < 0.001, **p < 0.01, *p < 0.05. **e**, AP-MS analysis of NP versus isotype control in A549 cells (WSN, MOI 3, 14 hpi), showing log2 fold changes of protein LFQ values for enrichment of NONO, NP and NS1 cross-linked partners and those not cross-linked. Data are derived from n = 3 independent biological replicates. Box plots as in (d). Statistical significance was assessed using a two-sided Wilcoxon rank-sum test. Significance levels: ***p < 0.001, **p < 0.01, *p < 0.05. **f,** Representative maximum projection of confocal images showing NEAT1_1 (green), vRNA (magenta), and DAPI (cyan) staining in mock and infected A549 cells at 4, 8, 12, and 24 hpi. Scale bar = 20 µm. **g,** CV of NEAT1_1 signal intensity and the number of paraspeckles per nucleus across different time points post-infection. n = 91–114 cells per condition. **h,** Maximum projection images of confocal images of lentiviral cell lines expressing mEGFP-tagged SFPQ, NONO, or PSPC1, and their colocalisation with NEAT1_2 (magenta). Scale bar = 20 µm. **i-k,** Line profiles of colocalisation: Gray value intensity profiles of NEAT1_2 (magenta) and mEGFP-tagged proteins (green) showing colocalisation across distance (in microns): (**i**) NONO, (**j**) SFPQ, and (**k**) PSPC1. **l-n,** Representative maximum projection of confocal images showing NEAT1_2 (magenta), vRNA (green), and DAPI (cyan) staining in mock and infected Calu-3 (**l**), MEFs (**m**), HBEpC (**n**) cells infected with WSN at 4, 8, 18, and 24 hpi. Scale bar = 20 µm. **o-p,** Representative maximum projection of confocal images showing NEAT1_2 (magenta), vRNA (green), and DAPI (cyan) staining in mock and infected A549 (**o**), HBEpC (**p**) cells infected with pdm09 at 4, 8, 18, and 24 hpi. Scale bar = 20 µm. **q-r,** Representative maximum projection of confocal images showing NEAT1_2 (magenta), vRNA (green), and DAPI (cyan) staining in mock and infected A549 (q), HBEpC (r) cells infected with H3N2/Aichi at 4, 8, 18, and 24 hpi. Scale bar = 20 µm.

s, CV of NEAT1_2 signal intensity and the number of paraspeckles per nucleus in A549 cells during pdm09 infection at various time points. n = 136–196 cells per condition. **t,** CV of NEAT1_2 signal intensity and the number of paraspeckles per nucleus in primary HBEpC cells during pdm09 infection at various time points. n = 134–393 cells per condition. **u,** CV of NEAT1_2 signal intensity and the number of paraspeckles per nucleus in A549 cells during H3N2/Aichi infection at various time points. n = 98–167 cells per condition. **v,** CV of NEAT1_2 signal intensity and the number of paraspeckles per nucleus in HBEpC cells during H3N2/Aichi infection at various time points. n = 148–379 cells per condition. **w,** Representative maximum projection of confocal images showing NEAT1_2 (magenta), vRNA (green), and DAPI (cyan) staining in mock and infected HeLa cells infected with WSN (MOI 3). Scale bar = 20 μm. **x,** CV of NEAT1_2 signal intensity and the number of paraspeckles per nucleus across different time points post-infection in HeLa cells. n = 39–167 cells per condition. **y,** Representative maximum projection of confocal images showing NEAT1_2 (magenta), mRNA (green) or GFP, and DAPI (cyan) staining in transfected HEK293T cells at 24 hpt. Scale bar = 20 μm. **z,** CV of NEAT1_2 signal intensity and the number of paraspeckles per nucleus across different time points post-infection in HEK293T cells. n = 12–30 cells per condition. All experiments in panels a–c, f, h, l–r, w and y were independently repeated at least twice with similar results; representative images and blots are shown. Data from panels (**g, s-v, x, z**) are shown as individual values. Statistical analysis was performed using one-way ANOVA with Dunnett's multiple comparisons test, two-sided. Significance levels: *p < 0.05, **p < 0.01, ***p < 0.001, *****p < 0.0001*. Exact P-values are provided in the Source Data.

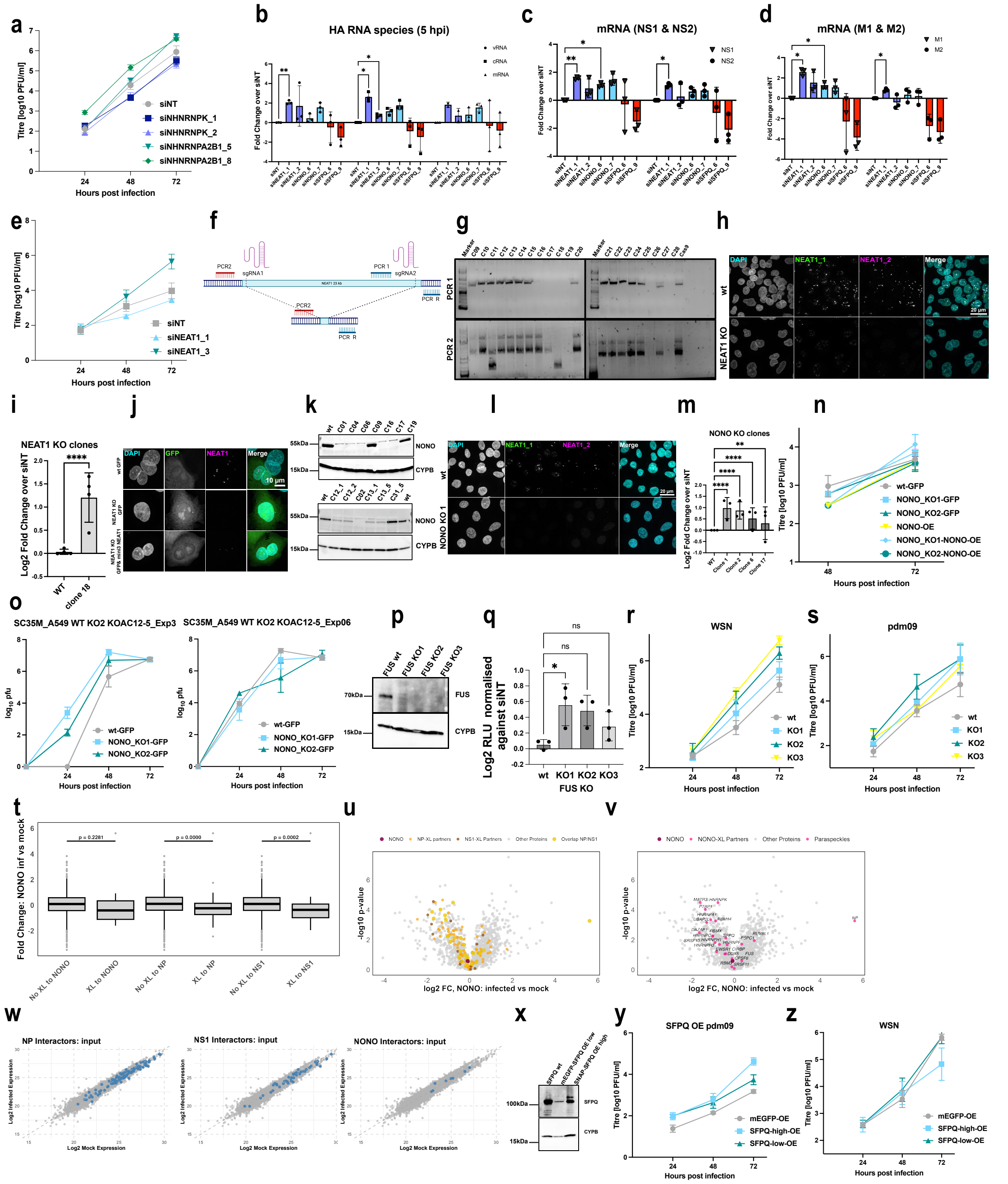

Supplementary Fig. 5 | Role of NEAT1 and NONO during IAV infection. a, Multi-cycle viral replication kinetics in A549 cells transfected with siRNAs targeting *HNRNPK* or *HNRNPA2B1* for 72 h prior to WSN infection (MOI 0.001). Data represent mean ± SD from n = 3 independent biological replicates (independent infections; unit of study), with titres measured at the indicated time points. P values (significant for *HNRNPK* at 48 and 74 hpi) are shown in Source Data. **b-d**, qPCR analysis of viral RNA species: HA fragment normalised to GAPDH (b); spliced and unspliced viral RNA species in A549 cells with knockdowns of paraspeckle proteins (WSN, MOI 3, 6 hpi): NS, NS2 (c) and M1, M2 (d). Data represent mean ± s.d. from n = 3 biological replicates (independent infections), each measured in duplicate (technical replicates). Technical replicates were averaged prior to statistical analysis. Statistical analysis was

performed using a two-sided mixed-effects model (REML) with Geisser–Greenhouse correction. Exact P values are provided in the source data. **e,** Multi-cycle viral replication kinetics in A549 cells transfected with siRNAs targeting *NEAT1* for 72 h prior to WSN infection (MOI 0.001). Data represent mean ± SD from n = 3 biological replicates (independent infections; unit of study), titres at the indicated time points. **f,** Strategy for *NEAT1* KO Generation: Schematic of the CRISPR/Cas9-based approach to generate *NEAT1* KO in A549 cells. Two sgRNAs are used to target *NEAT1*. PCR1 amplifies the region only in wild type (WT) or heterozygous clones, whereas PCR2 detects the shorter product (approximately 500 bp) in KO clones. For heterozygous clones, both the shorter KO product and the long (23 kb) WT product may appear. *Created in BioRender, https://BioRender.com/j78u936.* **g**, PCR Screening of Clones: Agarose gel showing PCR1 and PCR2 results for various clones. Clone 18 exhibits only the shorter PCR2 product, indicating a homozygous *NEAT1* KO, while Clone 11 shows both products, indicating heterozygosity. **h,** Maximum projection of confocal images of *NEAT1*: Representative images showing the localisation of NEAT1_1 (green) and NEAT1_2 (magenta) in WT and NEAT1 KO (Clone 28) cells. DAPI (cyan) counterstain nuclei. Scale bar = 20 µm. **i,** Quantification of luciferase activity in WT and *NEAT1* KO cells. Data represent mean ± SD from n = 4 independent biological replicates (independent infection performed on different days; unit of study). Each biological replicate consists of three technical replicates (wells), averaged prior to analysis. Two-way ANOVA. **j,** Overexpression of mini*NEAT1* 3 in A549 NEAT1 KO cells using electroporation partially rescued paraspeckle formation. **k,** Western blot showing *NONO* protein levels in WT and NONO KO clones generated using sgRNA1 (above) sgRNA2 (below). CYPB serves as a loading control. l, Maximum projection of confocal images of NEAT1: IF images of NEAT1_1 (green) and NEAT1_2 (magenta) in WT and *NONO* KO (Clone 2) cells. DAPI (cyan) counterstains nuclei. Scale bar = 20 µm. **m,** Quantification of luciferase activity in WT and *NONO* KO A549 cells. **n-o,** Multi-cycle viral replication kinetics in *NONO* KO A549 cells infected with pdm09 (MOI 0.001) (n) and avian SC35M (MOI 0.001) at early (Exp3) and late passage (Exp6) (o). Data are presented as mean ± SD from n = 3 independent biological replicates (independent infections), titres at indicated time points. **p**, Western blot showing *FUS* KO validation; CYPB as loading control. **q,** Luciferase assay in *FUS* KO A549 cells showing modest and variable increase in viral replication. **r–s,** Multi-cycle viral replication kinetics in *FUS* KO A549 cells infected with WSN (MOI 0.001) (r) and pdm09 (MOI 0.01) (s). Data are presented as mean ± SD from n = 3 independent biological replicates (independent infections), titres at indicated time points. **t,** Log2 fold change of protein LFQ values in NONO AP-MS (infected versus mock), including NONO, NP and NS1 cross-linked partners. Data are derived from n = 3 independent biological replicates (independent infections), each corresponding to one AP-MS experiment (unit of study). Box plots show the median (centre line), interquartile range (box), and 1.5× the interquartile range (whiskers); points outside this range are shown as individual outliers. Individual points represent proteins. Statistical significance was determined using a two-sided Wilcoxon rank-sum test on protein-level fold changes across replicates. **u, v,** Volcano plots showing log2 fold change of protein LFQ values versus statistical significance (-log10 P value, two-sided Student's t-test without multiple hypothesis correction, n=3 biological replicates) of NONO AP-MS in infected versus mock samples. XL-partners of NP or NS1 or both (overlap) are colour-coded as indicated (u) and paraspeckle proteins and NONO XL-partners are color-coded as indicated (v). **w,** Scatter plots of Log2 LFQ values comparing infected and mock input samples for AP-MS, with annotated XL-partners of proteins as indicated. **x,** Western

Blot depicting *SFPQ* overexpression in A549 cells. **y–z,** Multi-cycle viral growth curves in A549 cells overexpressing *SFPQ* constructs or control (mEGFP) infected with pdm09 (MOI 0.01) (y) and WSN (MOI 0.001) (z). Data are presented as mean ± SD from n = 3 independent biological replicates (independent infections), titres at indicated time points. All experiments in panels g, j, k, p and x were independently repeated at least twice with similar results; representative images and blots are shown. Data from panels (b-d, i, m, q) represent mean ± SD (n = 3). Statistical analysis was performed using mixed-effects model (b-d) or one-way ANOVA (i, m, q) with Dunnett's multiple comparisons test, two-sided. Significance levels: ***$p < 0.001$, **$p < 0.01$, *$p < 0.05$, ns = not significant. Exact P-values are provided in the Source Data.

Supplementary Tables

Supplementary Table 1 (Excel file) | Cross-linking data set. a, Cross-links filtered at 2% residue-pair FDR level. **b,** Protein pairs filtered at 2% protein-pair FDR level. **c,** Cross-linking data filtered at both 2% residue-pair and protein-pair FDR level. **d,** Virus-centric network containing cross-links to viral proteins and between host proteins that cross-linked to viral proteins. **e,** Cross-links filtered at 5% residue-pair FDR level. **f,** Protein pairs filtered at 5% protein-pair FDR level. **g,** Cross-linking data filtered at both 5% residue-pair and protein-pair FDR level.

Supplementary Table 2 (Excel file) | Overlap with previously reported protein–protein interactions. a, Protein pairs identified as PPIs in previous focused IAV studies. **b,** Host–host protein pairs identified in this study that are annotated in BioGRID as physical interactions.

Supplementary Table 3 (Excel file) | Functional enrichment analysis. a, Host proteins cross-linked to viral proteins and used for the enrichment analysis. **b,** Enriched Reactome Pathways. **c,** Enriched GO biological processes. **d,** Enriched GO cellular components. **e,** Enriched GO molecular functions. **f,** Proteins simultaneously enriched among the host proteins in the NONO AP-MS under mock conditions and NP AP-MS infected. See also Figure 7H. **g,** GO biological processes simultaneously enriched for proteins in **(f).** See also Figure 7I.

Supplementary Table 4 (Excel file) | Scores of structural models of IAV-human proteins pairs modelled using AlphaFold. a, Models built using AlphaFold 3. **b,** Models built using AF3x and all cross-links. **c,** Models built using AF3x and one cross-link at a time. d, Models built using AlphaFold 2. **e,** Selected models built using AlphaFold 3 run 1,000 times with different random generator seeds. **f,** Selected

models built using AF3x, one cross-link, and run 1,000 times with different random generator seeds. **g,** Models built using GRASP and all cross-links.

Supplementary Table 5 (Excel file) | FISH probe sequences. a, vRNA probes for WSN. b, vRNA probes for A/H3N2/Aichi. c, vRNA probes for A/pdm09. d, Viral mRNA probes for WSN. e, Viral cRNA probes for WSN. f, Mouse probes. g, NEAT1 probes.

Supplementary Table 6 (Excel file) | siRNAs used for knockdown experiments, all from Qiagen.

Supplementary Table 7 (Excel file) | qPCR primers. a, Human gene primers. b, Strand-specific qPCR primers for WSN viral RNA species. c, Viral gene primers.

Supplementary Table 8 (Excel file) | Antibodies used for immunoblotting and immunostaining.

Supplementary Video

Supplementary Video 1 | NONO or SFPQ cellular localisation in A549 cells stably overexpressing mEGFP-NONO or mEGFP-SFPQ infected with WSN at MOI 3 or mock-infected and imaged every 40 min

Supplementary References

(Eisfeld et al. 2015, 2011; Avalos et al. 1997; Veler et al. 2022; Böttcher-Friebertshäuser et al. 2013; Copeland et al. 1986; Ozawa et al. 2007; Dou et al. 2018; Manzoor et al. 2014; Jones et al. 1986; Huet et al. 2010; Graef et al. 2010; Long and Fodor 2016; Greenspan et al. 1988; Melén et al. 2007; Petrich et al. 2021; Sato et al. 2019)

Avalos, R. T., Z. Yu, and D. P. Nayak. 1997. 'Association of Influenza Virus NP and M1 Proteins with Cellular Cytoskeletal Elements in Influenza Virus-Infected Cells'. *Journal of Virology* 71 (4): 2947–58. https://doi.org/10.1128/JVI.71.4.2947-2958.1997.

Böttcher-Friebertshäuser, Eva, Hans-Dieter Klenk, and Wolfgang Garten. 2013. 'Activation of Influenza Viruses by Proteases from Host Cells and Bacteria in the Human Airway Epithelium'. *Pathogens and Disease* 69 (2): 87–100. https://doi.org/10.1111/2049-632X.12053.

Copeland, C. S., R. W. Doms, E. M. Bolzau, R. G. Webster, and A. Helenius. 1986. 'Assembly of Influenza Hemagglutinin Trimers and Its Role in Intracellular Transport'. *The Journal of Cell Biology* 103 (4): 1179–91. https://doi.org/10.1083/jcb.103.4.1179.

Dou, Dan, Rebecca Revol, Henrik Östbye, Hao Wang, and Robert Daniels. 2018. 'Influenza A Virus Cell Entry, Replication, Virion Assembly and Movement'. *Frontiers in Immunology* 9. https://www.frontiersin.org/article/10.3389/fimmu.2018.01581.

Eisfeld, Amie J., Eiryo Kawakami, Tokiko Watanabe, Gabriele Neumann, and Yoshihiro Kawaoka. 2011. 'RAB11A Is Essential for Transport of the Influenza Virus Genome to the Plasma Membrane'. *Journal of Virology* 85 (13): 6117–26. https://doi.org/10.1128/JVI.00378-11.

Eisfeld, Amie J., Gabriele Neumann, and Yoshihiro Kawaoka. 2015. 'At the Centre: Influenza A Virus Ribonucleoproteins'. *Nature Reviews Microbiology* 13 (1): 28–41. https://doi.org/10.1038/nrmicro3367.

Graef, Katy M., Frank T. Vreede, Yuk-Fai Lau, et al. 2010. 'The PB2 Subunit of the Influenza Virus RNA Polymerase Affects Virulence by Interacting with the Mitochondrial Antiviral Signaling Protein and Inhibiting Expression of Beta Interferon'. *Journal of Virology* 84 (17): 8433–45. https://doi.org/10.1128/jvi.00879-10.

Greenspan, D., P. Palese, and M. Krystal. 1988. 'Two Nuclear Location Signals in the Influenza Virus NS1 Nonstructural Protein'. *Journal of Virology* 62 (8): 3020–26. https://doi.org/10.1128/JVI.62.8.3020-3026.1988.

Huet, Sébastien, Sergiy V. Avilov, Lars Ferbitz, Nathalie Daigle, Stephen Cusack, and Jan Ellenberg. 2010. 'Nuclear Import and Assembly of Influenza A Virus RNA Polymerase Studied in Live Cells by Fluorescence Cross-Correlation

Spectroscopy'. *Journal of Virology* 84 (3): 1254–64. https://doi.org/10.1128/JVI.01533-09.

Jones, I. M., P. A. Reay, and K. L. Philpott. 1986. 'Nuclear Location of All Three Influenza Polymerase Proteins and a Nuclear Signal in Polymerase PB2.' *The EMBO Journal* 5 (9): 2371–76. https://doi.org/10.1002/j.1460-2075.1986.tb04506.x.

Long, Joshua C. D., and Ervin Fodor. 2016. 'The PB2 Subunit of the Influenza A Virus RNA Polymerase Is Imported into the Mitochondrial Matrix'. *Journal of Virology* 90 (19): 8729–38. https://doi.org/10.1128/JVI.01384-16.

Manzoor, Rashid, Kazumichi Kuroda, Reiko Yoshida, et al. 2014. 'Heat Shock Protein 70 Modulates Influenza A Virus Polymerase Activity*'. *Journal of Biological Chemistry* 289 (11): 7599–614. https://doi.org/10.1074/jbc.M113.507798.

Melén, Krister, Leena Kinnunen, Riku Fagerlund, et al. 2007. 'Nuclear and Nucleolar Targeting of Influenza A Virus NS1 Protein: Striking Differences between Different Virus Subtypes'. *Journal of Virology* 81 (11): 5995–6006. https://doi.org/10.1128/JVI.01714-06.

Ozawa, Makoto, Ken Fujii, Yukiko Muramoto, et al. 2007. 'Contributions of Two Nuclear Localization Signals of Influenza A Virus Nucleoprotein to Viral Replication'. *Journal of Virology* 81 (1): 30–41. https://doi.org/10.1128/JVI.01434-06.

Petrich, Annett, Valentin Dunsing, Sara Bobone, and Salvatore Chiantia. 2021. 'Influenza A M2 Recruits M1 to the Plasma Membrane: A Fluorescence Fluctuation Microscopy Study'. *Biophysical Journal* 120 (24): 5478–90. https://doi.org/10.1016/j.bpj.2021.11.023.

Sato, Ryota, Takashi Okura, Madoka Kawahara, Naoki Takizawa, Fumitaka Momose, and Yuko Morikawa. 2019. 'Apical Trafficking Pathways of Influenza A Virus HA and NA via Rab17- and Rab23-Positive Compartments'. *Frontiers in Microbiology* 10 (August). https://doi.org/10.3389/fmicb.2019.01857.

Veler, Hana, Haitian Fan, Jeremy R. Keown, et al. 2022. 'The C-Terminal Domains of the PB2 Subunit of the Influenza A Virus RNA Polymerase Directly Interact with Cellular GTPase Rab11a'. *Journal of Virology* 96 (5): e01979-21. https://doi.org/10.1128/jvi.01979-21.
