# Supplementary material for: Mapping in-cell protein contact sites reveals hijacking of paraspeckles during influenza A virus infection
Source: Nat Microbiol. 2026 Jul 20;11(8):2247–65. doi: 10.1038/s41564-026-02416-1 (PMC13423814; doi:10.1038/s41564-026-02416-1)
Supplement: Supplementary file 2 — Reporting Summary [file 41564_2026_2416_MOESM2_ESM.pdf]

nature portfolio

Corresponding author(s): Jan Kosinski, Boris Bogdanow, Fan Liu

Last updated by author(s): May 11, 2026

Reporting Summary

Nature Portfolio wishes to improve the reproducibility of the work that we publish. This form provides structure for consistency and transparency in reporting. For further information on Nature Portfolio policies, see our Editorial Policies and the Editorial Policy Checklist.

Statistics

For all statistical analyses, confirm that the following items are present in the figure legend, table legend, main text, or Methods section.

n/a	Confirmed	
☐	☒	The exact sample size (n) for each experimental group/condition, given as a discrete number and unit of measurement
☐	☒	A statement on whether measurements were taken from distinct samples or whether the same sample was measured repeatedly
☐	☒	The statistical test(s) used AND whether they are one- or two-sided<br>*Only common tests should be described solely by name; describe more complex techniques in the Methods section.*
☒	☐	A description of all covariates tested
☐	☒	A description of any assumptions or corrections, such as tests of normality and adjustment for multiple comparisons
☐	☒	A full description of the statistical parameters including central tendency (e.g. means) or other basic estimates (e.g. regression coefficient) AND variation (e.g. standard deviation) or associated estimates of uncertainty (e.g. confidence intervals)
☐	☒	For null hypothesis testing, the test statistic (e.g. F, t, r) with confidence intervals, effect sizes, degrees of freedom and P value noted<br>*Give P values as exact values whenever suitable.*
☒	☐	For Bayesian analysis, information on the choice of priors and Markov chain Monte Carlo settings
☒	☐	For hierarchical and complex designs, identification of the appropriate level for tests and full reporting of outcomes
☐	☒	Estimates of effect sizes (e.g. Cohen's d, Pearson's r), indicating how they were calculated

Our web collection on statistics for biologists contains articles on many of the points above.

Software and code

Policy information about availability of computer code

Data collection	MS: Peptides were analysed by LC–MS/MS on an Orbitrap Fusion Lumos mass spectrometer equipped with a FAIMS Pro Duo interface, using FAIMS compensation voltages of −50, −60, and −75V. Precursors with charge states +4 to +8 were fragmented by stepped higher-energy collisional dissociation, and MS2 spectra were acquired in the Orbitrap.<br>Glycoproteomics samples were TMT-labelled, pooled, pre-fractionated into 24 fractions, and analysed by LC–MS/MS on an Exploris 480.<br>Microscopy data were acquired using Nikon Elements software on a Nikon Ti2 microscope equipped with a CSU-W1 spinning disk confocal unit. Z-stacks were recorded.
Data analysis	Mass spectrometry data analysis: MaxQuant v1.6.2.6a, Scout v1.5.1, xiNET as of 2024<br>Glycoproteomics: raw files searched using MSFragger (v4.0) in FragPipe (v20.0) against combined human (SwissProt UP000005640) and Influenza A (UP000009255) databases.<br>Image analysis: Fiji (ImageJ v2.16.0/1.54p). CellProfiler v4.2.6<br>Structural analysis: AlphaFold version 2, AlphaFold version 3, AF3x #dfb94a3, AlphaPulldown version 2, UCSF ChimeraX 1.9, GRASP version 2a381bc.<br>Statistical analysis and plotting: GraphPad Prism 10 (used for qPCR, luciferase assays, and imaging quantifications)<br>Other bioinformatic analyses: Enrichr web platform as of December 2024

For manuscripts utilizing custom algorithms or software that are central to the research but not yet described in published literature, software must be made available to editors and reviewers. We strongly encourage code deposition in a community repository (e.g. GitHub). See the Nature Portfolio guidelines for submitting code & software for further information.

Data

Policy information about availability of data

All manuscripts must include a data availability statement. This statement should provide the following information, where applicable:

- Accession codes, unique identifiers, or web links for publicly available datasets
- A description of any restrictions on data availability
- For clinical datasets or third party data, please ensure that the statement adheres to our policy

Structural models have been deposited on Zenodo under the under the following URL: https://doi.org/10.5281/zenodo.18053782. Fluorescent images will be deposited in the BioImage Archive. The mass spectrometry cross-linking data have been deposited to the ProteomeXchange Consortium via the PRIDE partner repository with the dataset identifier PXD071226 (reviewer username: reviewer_pxd071226@ebi.ac.uk, password: 3nAft4fE7hGu). The mass spectrometry glycoproteomics data have been deposited to the ProteomeXchange Consortium via the PRIDE partner repository with the dataset identifier PXD072851 (Username: reviewer_pxd072851@ebi.ac.uk, Password: gVfUOEDHOakB).

Research involving human participants, their data, or biological material

Policy information about studies with human participants or human data. See also policy information about sex, gender (identity/presentation), and sexual orientation and race, ethnicity and racism.

Reporting on sex and gender	*Use the terms sex (biological attribute) and gender (shaped by social and cultural circumstances) carefully in order to avoid confusing both terms. Indicate if findings apply to only one sex or gender; describe whether sex and gender were considered in study design; whether sex and/or gender was determined based on self-reporting or assigned and methods used. Provide in the source data disaggregated sex and gender data, where this information has been collected, and if consent has been obtained for sharing of individual-level data; provide overall numbers in this Reporting Summary. Please state if this information has not been collected. Report sex- and gender-based analyses where performed, justify reasons for lack of sex- and gender-based analysis.*
Reporting on race, ethnicity, or other socially relevant groupings	*Please specify the socially constructed or socially relevant categorization variable(s) used in your manuscript and explain why they were used. Please note that such variables should not be used as proxies for other socially constructed/relevant variables (for example, race or ethnicity should not be used as a proxy for socioeconomic status). Provide clear definitions of the relevant terms used, how they were provided (by the participants/respondents, the researchers, or third parties), and the method(s) used to classify people into the different categories (e.g. self-report, census or administrative data, social media data, etc.) Please provide details about how you controlled for confounding variables in your analyses.*
Population characteristics	*Describe the covariate-relevant population characteristics of the human research participants (e.g. age, genotypic information, past and current diagnosis and treatment categories). If you filled out the behavioural & social sciences study design questions and have nothing to add here, write "See above."*
Recruitment	*Describe how participants were recruited. Outline any potential self-selection bias or other biases that may be present and how these are likely to impact results.*
Ethics oversight	*Identify the organization(s) that approved the study protocol.*

Note that full information on the approval of the study protocol must also be provided in the manuscript.

Field-specific reporting

Please select the one below that is the best fit for your research. If you are not sure, read the appropriate sections before making your selection.

☒ Life sciences ☐ Behavioural & social sciences ☐ Ecological, evolutionary & environmental sciences

For a reference copy of the document with all sections, see nature.com/documents/nr-reporting-summary-flat.pdf

Life sciences study design

All studies must disclose on these points even when the disclosure is negative.

Sample size	Sample sizes were based on standard practice in the field and are consistent with similar published studies. No statistical method was used to predetermine sample size. For quantitative assays, a minimum of three independent biological replicates were used unless otherwise stated. Sample size was sufficient to observe consistent and statistically supported effects across experiments.
Data exclusions	No data were excluded from the analysis unless clearly justified due to technical failure (e.g., unsuccessful transfection or infection). In qPCR experiments, data points showing clear signs of contamination (e.g., abnormally high amplification curves or melt curve anomalies) were excluded from analysis; however, all raw values were included in the source data file, and any exclusions were transparently annotated. Exclusion criteria were defined prior to analysis and applied consistently.
Replication	All key experiments were performed in at least three independent biological replicates. Where applicable (e.g., qPCR, luciferase assays,

Replication	imaging), each biological replicate included technical duplicates or triplicates. All findings reported were reproducible.
Randomization	All light microscopy data comes from randomly selected cells. Experimental groups (e.g., siRNA treatments, virus-infected vs. mock) were defined by experimental design rather than random allocation.
Blinding	Data collection and analysis were performed by the same investigators who were aware of the experimental conditions, as group allocation (e.g., infection status, genetic modification) was inherently linked to the methodology and necessary for interpretation. However, image analysis was fully automated and conducted identically across all conditions using the same parameters.

Reporting for specific materials, systems and methods

We require information from authors about some types of materials, experimental systems and methods used in many studies. Here, indicate whether each material, system or method listed is relevant to your study. If you are not sure if a list item applies to your research, read the appropriate section before selecting a response.

Materials & experimental systems

n/a	Involved in the study	
☐	☒	Antibodies
☐	☒	Eukaryotic cell lines
☒	☐	Palaeontology and archaeology
☒	☐	Animals and other organisms
☒	☐	Clinical data
☒	☐	Dual use research of concern
☒	☐	Plants

Methods

n/a	Involved in the study	
☒	☐	ChIP-seq
☒	☐	Flow cytometry
☒	☐	MRI-based neuroimaging

Antibodies

Antibodies used

Mouse monoclonal anti-NS1 IAV H1N1 (Clone GT1653), Thermo Fisher Scientific, Cat# MA5-35909
Mouse monoclonal anti-NONO (Clone 2A2B10), Proteintech, Cat# 66361-1-Ig
Mouse monoclonal anti-SFPQ (Clone 1G4A5), Proteintech, Cat# 67129-1-Ig
Mouse monoclonal anti-IAV NP (Clone C43), Abcam, Cat# ab128193
Mouse monoclonal anti-Influenza A M2 (Clone 14C2), Thermo Fisher Scientific, Cat# MA1-082
Rabbit polyclonal anti-NONO, Proteintech, Cat# 11058-1-AP
Rabbit polyclonal anti-SLC7A5, Proteintech, Cat# 28670-1-AP
Rabbit polyclonal anti-SLC3A2, Proteintech, Cat# 15193-1-AP
Rabbit monoclonal anti-Influenza A NP (Clone HL1089), Thermo Fisher Scientific, Cat# MA5-42364
Mouse monoclonal anti-SLC7A5 (Clone 2G5H3), Proteintech, Cat# 67951-1-Ig
Mouse monoclonal anti-SLC3A2 (Clone 2B10F5), Proteintech, Cat# 66883-1-Ig
Mouse monoclonal anti-IAV NP (Clone 9G8), Abcam, Cat# ab43821
Rabbit polyclonal anti-PSPC1, Proteintech, Cat# 16714-1-AP
Rabbit Gamma Globulin, Thermo Fisher Scientific, Cat# 31887
Mouse Gamma Globulin, Thermo Fisher Scientific, Cat# 31878
Rabbit monoclonal anti-Cyclophilin B (Clone D1V5J), Cell Signaling Technology, Cat# 43603
Mouse monoclonal anti-Vinculin (Clone hVIN-1), Merck, Cat# V9131
Rabbit polyclonal anti-Influenza A PB1, Thermo Fisher Scientific, Cat# PA5-34914
Rabbit polyclonal anti-Influenza A PB2, Thermo Fisher Scientific, Cat# PA5-32220
Mouse monoclonal anti-Influenza A M1 (Clone GA2B), Abcam, Cat# ab22396
Mouse anti-Influenza A virus nucleoprotein (NP) (clone C43, Abcam),
rabbit anti-NS1 (EPR28247-51, Abcam),
mouse anti-HA (clone 1.B.408, Abcam),
rabbit anti-PB2 (PA5-32220, Thermo Fisher Scientific),
mouse anti-M1 (clone GA2B, MA1-80736, Thermo Fisher Scientific),
mouse anti-HNRNPK (F45P9C7, Thermo Fisher Scientific),
mouse anti-RAB11A (67902-1-Ig, Proteintech),
mouse anti-HNRNPA1 (67844-1-Ig, Proteintech),
mouse anti-NF45 (67685-1-Ig, Proteintech),
mouse anti-HNRNPA2B1 (67445-1-Ig, Proteintech),
mouse anti-CCAR2 (66497-1-Ig, Proteintech),
mouse anti-SFPQ (67129-1-Ig, Proteintech),
rabbit anti-B4GALT1 (PA5-52744, Thermo Fisher Scientific),
rabbit anti-VIP36 (PA5-90437, Thermo Fisher Scientific),
rabbit anti-HNRNPM (26897-1-Ig, Proteintech),
rabbit anti-ERP29 (24344-1-AP, Proteintech),
rabbit anti-RPL35 (14826-1-AP, Proteintech),
rabbit anti-ERGIC-53 (13364-1-AP, Proteintech),
rabbit anti-HNRNPC (11760-1-AP, Proteintech),
rabbit anti-ANP32B (10843-1-AP, Proteintech).

Validation

All antibodies used in this study were commercially sourced and selected based on manufacturer validation for the species and

Validation

application (WB, IP, PLA or IF), as listed on respective product pages. For immunoprecipitation and pull-down experiments, positive and negative controls (e.g. isotype controls, tag-only controls, or knockdown/knockout conditions) were used to ensure specificity.

Eukaryotic cell lines

Policy information about cell lines and Sex and Gender in Research

Cell line source(s)

A549 (ECACC 86012804), HEK293T (ATCC CRL-3216), Calu-3 (provided by P. Chlanda, Heidelberg University; originally from Prof. R. Bartenschlager, Heidelberg University), MDCK (ECACC 84121903), MDCK.II (ECACC 00062107), MDCK-SIAT1 (Sigma-Aldrich 05071502-1VL), MDBK (provided by E. Fodor, University of Oxford; Sir William Dunn School of Pathology Cell Bank stock), MEFs (provided by Prof. Dr. Michael Bader, Max Delbrück Center, Berlin), HeLa Kyoto (provided by the Mahamid laboratory; originally from the Sara Cuylen-Haering laboratory and Daniel Gerlich, IMBA, Vienna, Austria; RRID:CVCL_1922), and primary HBEpCs (PromoCell C-12640, donor lot #499Z012.1) were used in this study. CRISPR-modified A549 cell lines (ΔNEAT1, ΔNONO, and ΔFUS) and lentiviral mEGFP-tagged A549 cell lines expressing NONO, PSPC1, or SFPQ were generated in this study.

Authentication

A549, HEK293T, MDCK, MDCK.II cells were obtained from ECACC, and primary HBEpCs were obtained from PromoCell which provides authenticated cell lines or primary cellsHeLa Kyoto stock as originally obtained from S. Narumiya, Kyoto University, Japan, RRID:CVCL_1922, and authenticated by Multiplex human Cell line Authentication testing. in case of HBEpCs. Calu-3, MEFs, and MDBK cells were obtained from collaborators and were not independently authenticated in our laboratory. A549 and HEK293T cells were authenticated by short tandem repeat (STR) profiling. CRISPR-edited A549 lines were generated from authenticated parental A549 cells. Knockouts were confirmed by qPCR and genomic PCR for long non-coding RNAs and by Western blot for protein-coding genes.

Mycoplasma contamination

All cell lines used in this study were routinely tested for mycoplasma contamination using the Mycoplasma Testing for Cell Culture service provided by Microsynth. Heat-inactivated samples were sent to the provider, who performed the extraction and testing. All cell lines tested negative prior to use in experiments.

Commonly misidentified lines
(See ICLAC register)

HEK293T and HeLa Kyoto cells appear in the ICLAC Register of Misidentified Cell Lines due to the historical HeLa cross-contamination risk. HEK293T was used only in non-infectious contexts (virus stock production, overexpression and pull-downs) and was not STR-authenticated. HeLa Kyoto cells were validated by Multiplex human Cell line Authentication testing. All cell lines were routinely tested for mycoplasma contamination and confirmed negative.

Plants

Seed stocks

Report on the source of all seed stocks or other plant material used. If applicable, state the seed stock centre and catalogue number. If plant specimens were collected from the field, describe the collection location, date and sampling procedures.

Novel plant genotypes

Describe the methods by which all novel plant genotypes were produced. This includes those generated by transgenic approaches, gene editing, chemical/radiation-based mutagenesis and hybridization. For transgenic lines, describe the transformation method, the number of independent lines analyzed and the generation upon which experiments were performed. For gene-edited lines, describe the editor used, the endogenous sequence targeted for editing, the targeting guide RNA sequence (if applicable) and how the editor was applied.

Authentication

Describe any authentication procedures for each seed stock used or novel genotype generated. Describe any experiments used to assess the effect of a mutation and, where applicable, how potential secondary effects (e.g. second site T-DNA insertions, mosiacism, off-target gene editing) were examined.
